# CBD Effects on Motor Profile and Neurobiological Indices Related to Glutamatergic Function Induced by Repeated Ketamine Pre-Administration

**DOI:** 10.3389/fphar.2021.746935

**Published:** 2021-10-27

**Authors:** Nafsika Poulia, Foteini Delis, Charalampos Brakatselos, George Ntoulas, Michail-Zois Asprogerakas, Katerina Antoniou

**Affiliations:** Department of Pharmacology, University of Ioannina, Ioannina, Greece

**Keywords:** ketamine, cannabidiol, rats, NMDA, AMPA, P-ERK1/2, motor activity

## Abstract

Clinical evidence and experimental studies have shown the psychotomimetic properties induced by ketamine. Moreover, acute or chronic ketamine (KET) administration has been widely used for modeling schizophrenia-like symptomatology and pathophysiology. Several studies have reported the antipsychotic potential of cannabidiol (CBD), while there is limited information on the cannabidiol effect on KET-induced schizophrenia-like impairments. Therefore, the goal of the present study was to evaluate neuroplastic changes induced by repeated KET administration, which is used as an experimental model of schizophrenia—with a behavioral focus on positive-like symptomatology– and to assess the modulatory role of CBD treatment. The present findings have shown a robust increase in motor activity in KET-treated rats, following a 10-day period of chronic administration at the sub-anesthetic dose of 30 mg/kg (i.p), that was reversed to normal by subsequent chronic CBD treatment. Concerning the expression of glutamate receptors, the current findings have shown region-dependent KET-induced constitutional alterations in NMDA and AMPA receptors that were modified by subsequent CBD treatment. Additionally, repeated KET administration increased ERK1/2 phosphorylation state in all regions examined, apart from the ventral hippocampus that was modulated by subsequent CBD treatment. The present results show, for the first time, a stimulated motor output coupled with a specific glutamatergic-related status and ERK1/2 activation following chronic KET administration that were attenuated by CBD treatment, in a region-dependent manner. These findings provide novel information concerning the antipsychotic potential of CBD using a specific design of chronic KET administration, thus contributing to experimental approaches that mirror the symptomatology and pathophysiology of schizophrenia.

## Introduction

Ketamine (KET) has been extensively used as an anesthetic, analgesic, and recently as an antidepressant, whereas each of the aforementioned indications has been related to a distinct dose range (Nowacka and Borczyk, 2019). Moreover, clinical and preclinical studies have shown the psychotomimetic KET-induced profile (Duncan et al., 1998; Georgiadou et al., 2014; Brakatselos et al., 2021) and, as a result, acute and chronic sub-anesthetic KET administration has been widely used to model psychosis and schizophrenia-like symptomatology and pathophysiology (Malhotra et al., 1997; Lahti et al., 2001; Becker et al., 2003; Nikiforuk and Popik, 2012; Beck et al., 2020; Kozela et al., 2020).

Ketamine acts as an uncompetitive inhibitor of NMDA glutamate receptors (Martin and Lodge, 1985). NMDA receptor hypofunction is associated with the neurobiology and symptomatology of schizophrenia, while glutamatergic neurotransmission has been essentially related to cognitive function and neuroplasticity (Frohlich and Van Horn, 2014; Piva et al., 2021).

Post-mortem findings from patients with schizophrenia, as well as preclinical studies link the disease’s biological substrate with impaired expression of glutamate receptors (Meador-Woodruff and Healy, 2000; Mueller et al., 2004; Benesh et al., 2020), which, in turn, is regarded as an index of neuroplasticity (El-Gaamouch et al., 2012; Brakatselos et al., 2021; Piva et al., 2021). In parallel, NMDA receptor inhibitors, including KET, have been shown to affect neuroplastic indices in a plethora of experimental models; these neuroplastic effects involve changes in the glutamate receptor status in terms of expression, subunit composition, phosphorylation state, and downstream signaling (Anver et al., 2011; Kamiyama et al., 2011; McNally et al., 2011; Izumi and Zorumski, 2014; Widman and McMahon, 2018; Piva et al., 2021). In particular, ERK1/2 signaling is involved in NMDA receptors downstream processes, and plays a pivotal role in the integration of neurotransmission events, the regulation of synaptic plasticity, and cognitive functions (Sun and Nan 2017; Albert-Gascó et al., 2020), while preclinical and post-mortem studies associate abnormal ERK signaling with schizophrenia manifestation (Kyosseva 2003). Furthermore, KET has been shown to affect ERK signaling in brain regions associated with the biological substrate of schizophrenia (Ahn et al., 2006; Bharne et al., 2016; Nygard et al., 2017; Meng et al., 2020). Although there are many studies investigating the effects of acute or repeated KET administration (Becker et al., 2003; Trujillo et al., 2008; Nikiforuk and Popik, 2012; Sampaio et al., 2018), there is no consensus on a specific experimental pattern for approaching schizophrenia and linking symptomatology to neurobiology, while there is limited information concerning the impact of acute or repeated KET administration on neuroplasticity indices (Luo et al., 2020; Brakatselos et al., 2021; Piva et al., 2021).

Clinical studies have shown that cannabidiol (CBD), a non-addictive cannabis compound, ameliorates schizophrenia symptoms (Leweke et al., 2012; Leweke et al., 2016; Rohleder et al., 2016; McGuire et al., 2018). Several experimental studies support the anti-psychotic properties of CBD by investigating its potential to counteract/reverse behavioral and neurobiological abnormalities that mirror psychosis and schizophrenia (Long et al., 2012; Rohleder et al., 2016; Osborne et al., 2017; Hudson et al., 2019; Rodrigues da Silva et al., 2020). CBD has been also reported to modulate acute KET-induced effects on neurobiological neuroplastic indices (Brakatselos et al., 2021), while limited data have been published regarding the effect of CBD on KET-induced schizophrenia-like impairments (Kozela et al., 2020).

Thus, in the present exploratory study first we aimed to define a repeated KET administration protocol that produces robust hyperlocomotion, a behavioral performance that has been widely used for psychosis modeling and evaluation of antipsychotic potential. Moreover, we focused to readout the glutamatergic status induced by KET-administrations, by estimating the protein expression levels of specific glutamate receptors, since ketamine is an uncompetitive NMDA inhibitor. In addition, we aimed to investigate the modulatory role of cannabidiol on the behavioral profile and the expression of glutamate receptors. In particular, our rationale includes the impact of CBD on KET-induced expression of NMDA and AMPA receptor subunits along with ERK1/2 phosphorylation state, in distinct rat brain regions involved in the pathophysiology of schizophrenia, such as the prefrontal cortex (PFC), nucleus accumbens (NAc), dorsal and ventral hippocampus (DH, VH).

## Materials and Methods

### Animals

Male 3-month-old Sprague-Dawley rats (*n* = 81) raised in the Animal Facility of the University of Ioannina (license № “EL33-BIObr01”) were housed in plastic cages (47.5 cm length × 20.5 cm height × 27 cm width), two per cage. The animals were maintained in a climate-regulated environment, under a 12-h light/dark cycle (lights on at 7:00 AM), with free access to food and water. All experiments took place during the light phase of the cycle.

All experiments were approved by the Institutional Animal Facility Committee of the University of Ioannina and performed in accordance with the Council Directive 2010/63/EU of the European Parliament and the Council of 22 September 2010 on the protection of animals used for scientific purposes.

### Drugs

Ketamine (Ketamidor®) was dissolved in saline (0.9%NaCl, sterile). CBD (>99% pure) was prepared according to previously established protocols (Brakatselos et al., 2021; Tzimas et al*.*, 2021). CBD was isolated from fiber-type *C. sativa* inflorescences of an EU-approved variety (‘Futura 75’). The final CBD product (>99% pure) was dissolved in vehicle solution (5% dimethylsulfoxide, 5% cremophor EL, sterile saline). All drugs were injected intraperitoneally (i.p.). All drugs were injected at the volume of 1 ml/kg.

### Experimental Design

Experimental groups were formed as below: Thirteen-week-old rats received 30 mg/kg KET or SAL once daily for 7 or 10 days, followed by a 2-days washout period. Next, they were injected with CBD (10 mg/kg, i.p.) or VEH, once daily for 5 days. Three days after the last CBD injection, the rats were either introduced to the open-field apparatus and their motor activity was recorded for 1 h or they were euthanized and their brains were immediately removed over ice for western blot analysis.

### Behavioral Analysis

All animals were accustomed to the experimental room for 1 week before the initiation of the experimental procedures and were handled twice daily for 3 days prior to commencement of testing. All animals were accustomed to the experimental room for 40 min prior to the experiments.

Motor behavior was recorded with a computerized activity monitoring system (ENV515, Activity Monitor, version 5; Med Associates Inc., United States) in a transparent, cubic open-field apparatus (40 cm × 40 cm x 40 cm). Animal ambulatory activity was recorded for 1 h. Activity during the first 15 min is considered a reaction to novelty (Galanopoulos et al., 2011; Pitsikas et al., 2019; Poulia et al., 2021). The rats are considered habituated to the open-field apparatus during the last 45 min of the test.

### Ketamine-Induced Activity Profile and Cannabidiol Potential Counteracting Role

Rats were injected with sub-anesthetic KET (30 mg/kg, i. p.) or saline (SAL) in their home cages once daily for 7 days. On 20th day, the rats were placed in the open-field apparatus and their motor activity was recorded for 1 h.

Different groups of rats were injected with KET (30 mg/kg, i.p.) or saline in their home cages once daily for 10 days. On the 20th day, the rats were placed in the open-field apparatus and their motor activity was recorded for 1 h.

Based on our results obtained from the two aforementioned treatment protocols, we chose to further proceed with behavioral and neurobiological evaluations, using the repeated ketamine protocol, namely the 10-days ketamine administration, since this protocol showed more robust effects on the behavioral outcomes.

The potential counteracting effects of CBD were investigated, and another subset of rats was used. Specifically, rats were injected with KET (30 mg/kg, i.p.) or saline in their home cages once daily for 10 days followed by a washout period (2 days). Next, they were injected with CBD (10 mg/kg, i.p.) or vehicle, once daily for 5 days. Three days after the last CBD injection, the rats were introduced to the open-field apparatus and their motor activity was recorded for 1 hour.

Each treatment group was consisted of 10–14 rats. In particular, SAL/VEH: 14 rats, KETx7: 14 rats, KETx10: 11 rats (1 missing value), SAL/CBD: 12 rats, KET/CBD: 10 rats (2 missing values). The selected dose of KET (30 mg/kg) was based on our pilot experiments and previous studies which have shown that this dose is the appropriate for KET-induced schizophrenia-like model (Becker et al., 2003; Becker et al., 2009; Nikiforuk and Popik 2012; Kozela et al., 2020). CBD dose was selected based on our acute studies (Brakatselos et al., 2021), and our preliminary data which are in line with a recent study where CBD counteracted repeated KET (30 mg/kg)-induced effects on cognitive functions and transcriptomic alterations (Kozela et al., 2020). Moreover, the duration of the wash-out periods was chosen based on the aforementioned studies, and reports regarding KET (Zanos et al., 2018) and CBD (Hložek et al., 2017) pharmacokinetic properties.

### Western Blot

Adult rats were used to assess the effects of CBD on 10-days KET-induced protein expression. Specifically, rats received KET (30 mg/kg, i.p.) or saline in their home cages once daily for 10 days followed by a washout period (2 days). Next, they were injected with CBD (10 mg/kg, i.p.) or vehicle once daily for 5 days. Three days after the last CBD injection, the rats were anesthetized with isoflurane, euthanized by decapitation and their brains were immediately removed over ice. Dissected prefrontal cortex, nucleus accumbens, dorsal hippocampus, and ventral hippocampus were then homogenized as previously described (Brakatselos et al., 2021; Poulia et al., 2021) with minor modifications.

Briefly, tissues were homogenized with RIPA buffer containing phosphatase and protease inhibitors. Protein lysates were diluted in Laemli loading buffer and heated at 95°C for 5 min. After electrophoresis, gels were semi-dry transferred onto a nitrocellulose membrane. Membranes were blocked in 5% non-fat milk/TBS-Tween 0.1% for 1 h at room temperature and incubated overnight at 4°C with one of the following primary antibodies: Anti-NMDA R1 (GluN1) (1:1,000, rabbit mAb, Cell Signaling 5704), anti-NMDA R2Α (GluN2Α) (1:1,000, rabbit mAb, Cell Signaling 4205), anti-NMDA R2B (GluN2B) (1:1,000, rabbit mAb, Cell Signaling 14544), anti-AMPA R1 (GluA1) (1:2,000, rabbit mAb Cell Signaling 13185), anti-AMPA R2 (GluA2) (1:2000, rabbit mAb, Cell Signalling 13607), anti-p44/42 MAPK (Erk1/2) (1:5,000, rabbit mAb, Cell Signaling 4695) and anti-phospho-p44/42 MAPK (Erk1/2) (Thr202/Tyr204) (1:5,000, rabbit Ab, Cell Signaling 9101).

Next, membranes were incubated for 1 h at room temperature with an HRP-linked secondary antibody (1:5000, goat anti-rabbit IgG HRP-conjugate, Merck Millipore #12-348). Every sample was standardized with *α*-tubulin (1:4,000, rabbit mAb, Cell Signaling 2144).

Immunoreactive bands were visualized with enhanced chemiluminescence detection solutions (Biorad, Clarity™ Western ECL Blotting Substrates) using an XRS charge-coupled device camera (Bio-Rad Laboratories) and Quantity One software. The optical density of each protein band was normalized over the corresponding loading marker band (*α*-tubulin). Optical densities were quantified with ImageJ software.

To estimate the proportion of phosphorylated ERK1/2 (p-ERK1/2) over total ERK (t-ERK), the western blot analysis was executed with duplicated membranes; one was used for the detection of p-ERK1/2 and the other for the detection of total ERK1/2 levels. The ratio of the two normalized optical densities was calculated for each sample to determine the p-ERK/t-ERK ratio.

### Statistical Analysis

Effects of repeated KET administration on open-field activity (ambulatory distance) were analyzed using one-way ANOVA, with Bonferroni post-hoc test for multiple comparisons. Effects of CBD treatment after KET pre-administration on motor activity and neurobiological indices were analyzed using two-way ANOVA with Bonferroni post-hoc test for multiple comparisons. Analysis was performed with SPSS v.21 (IBM Corp, NY, United States). Data are presented as mean ± SD. Overall level of significance was set at *p* < 0.05.

## Results

**FIGURE 1:**
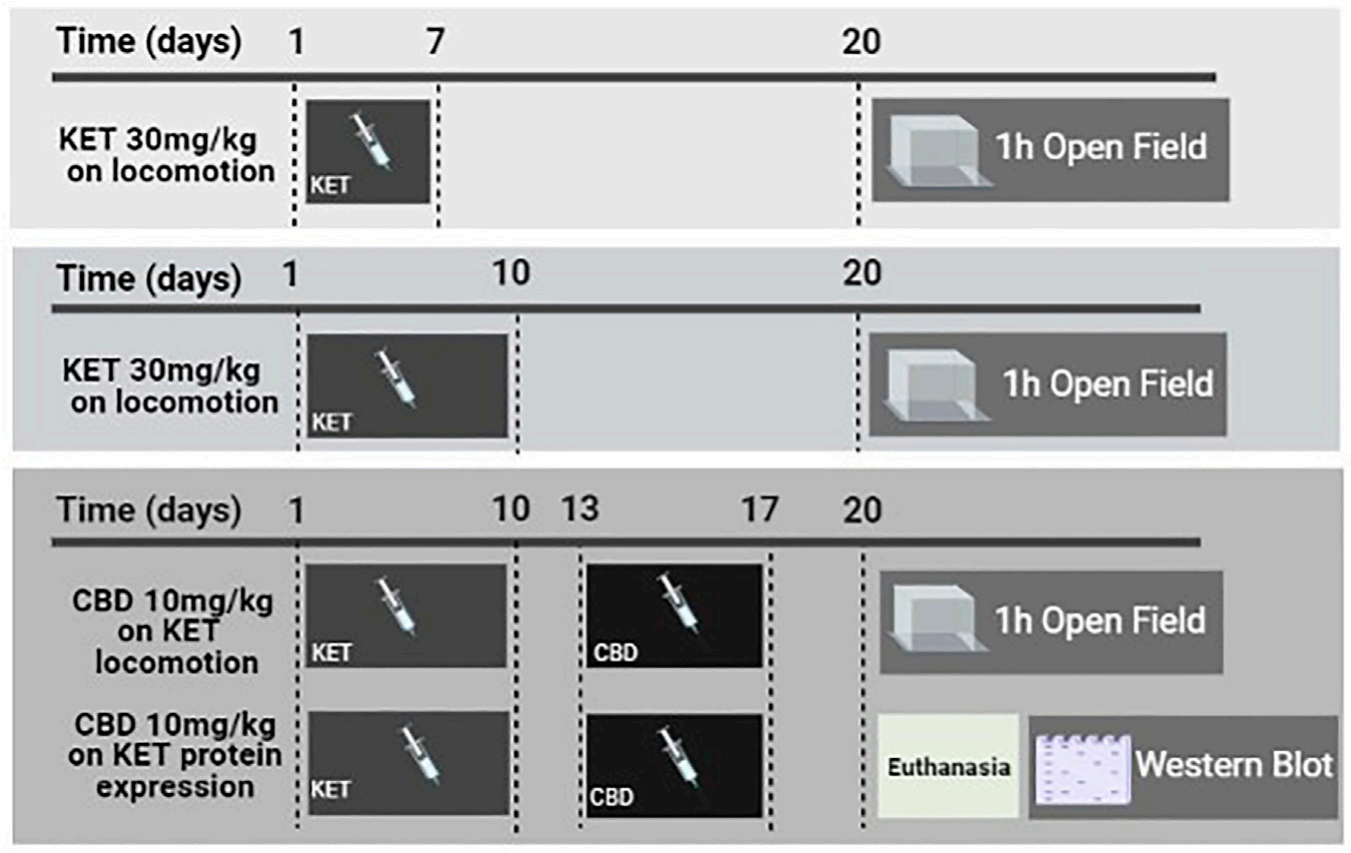
Schematic presentation of the experimental timeline depicting the procedures implemented in this study. KET and CBD refer to ketamine and cannabidiol, respectively.

The timeline of the experimental protocol is presented in Figure 1.

### Behavioral Analysis

#### Repeated Ketamine Administration Increased Motor Activity

**FIGURE 2:**
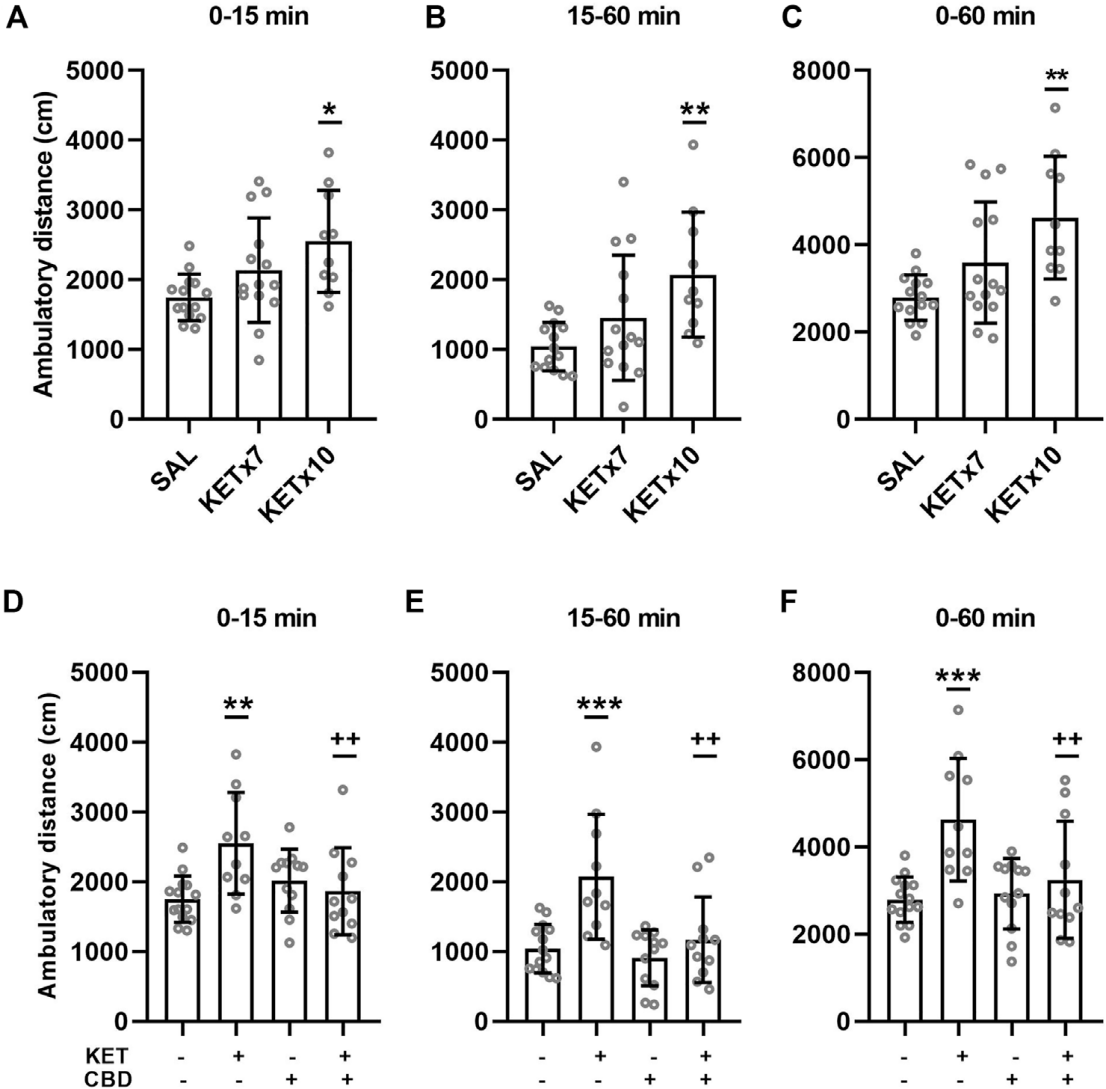
**(A–C)**. Effects of 7-days and 10-days ketamine (KET) administration on horizontal, ambulatory activity in the open-field chamber. The values represent the mean ± SD (*n* = 10–14 animals per group). Statistical analysis was performed by one-way ANOVA (Bonferroni’s post hoc test). *p < 0.05, **p < 0.01 vs saline (SAL). **(D–F)**. Effects of cannabidiol (CBD) administration on horizontal, ambulatory activity in the open-field chamber, after 10-days KET pretreatment. The values represent the mean ± SD (*n* = 10–14 animals per group). Statistical analysis was performed by two-way ANOVA (Bonferroni’s post hoc test). **p < 0.01, ***p < 0.001 vs SAL/VEH; ++*p*< 0.01 vs KET/VEH.

Effects of repeated KET administration on horizontal, ambulatory activity are presented in Figure 2A–C.

One-way ANOVA revealed an effect of KET treatment on ambulatory distance during the first 15 min of the open-field test [F_(2,35)_ = 4.89, *p* = 0.013]. Post-hoc comparisons revealed an increase in spontaneous ambulation following chronic (x10 days) KET administration compared to control (*p* = 0.010). Seven-day KET administration did not affect spontaneous ambulation compared to control.

One-way ANOVA revealed an effect of KET treatment on ambulatory distance during the last 45 min of the open-field test (15–60 min) [F_(2,35)_ = 5.64, *p* = 0.008]. Post-hoc comparisons revealed an increase in habituated ambulation following 10-days KET administration compared to control (*p* = 0.006). Seven-day KET administration did not affect habituated ambulation compared to control.

One-way ANOVA revealed an effect of KET treatment ambulatory distance during the entire open-field test (0–60 min) [F_(2,35)_ = 7.38, *p* = 0.002]. Post-hoc comparisons revealed an increase in total ambulatory distance following chronic (x10 days) KET administration compared to control (*p* = 0.001). Repeated 7-days KET administration did not affect total ambulation, compared to control.

#### Cannabidiol Treatment Reversed KET-Induced Hyperactivity

Effects of CBD on KET-induced horizontal hyperactivity are presented in Figure 2D–F.

A significant KET × CBD interaction [F_(1,43)_ = 9.19, *p* = 0.004] was observed for ambulatory distance during the first 15 min of the test (spontaneous, response to novelty). Ambulatory distance was significantly higher in KET/VEH rats, compared to SAL/VEH (*p* = 0.001) and KET/CBD (*p* = 0.005), while it remained unaffected in SAL/CBD rats.

A significant KET × CBD interaction [F_(1,43)_ = 5.22, *p* = 0.027] was observed for ambulatory distance during the last 45 min of the open-field test (15–60 min, habituated rats). Ambulatory distance was significantly higher in KET/VEH rats, compared to SAL/VEH (*p* < 0.001) and KET/CBD (*p* = 0.001), while it remained unaffected in SAL/CBD rats.

A significant KET × CBD interaction [F_(1,43)_ = 4.11, *p* = 0.049] was also observed for ambulatory distance during the entire 60 min of the open-field test. Ambulatory distance was significantly higher in KET/VEH rats, compared to SAL/VEH (*p* < 0.001) and KET/CBD (*p* = 0.004), while it remained unaffected in SAL/CBD rats.

### Western Blot Analysis

CBD treatment reversed KET-induced effects on NMDA receptor subunit expression, in specific rat brain regions.

**FIGURE 3:**
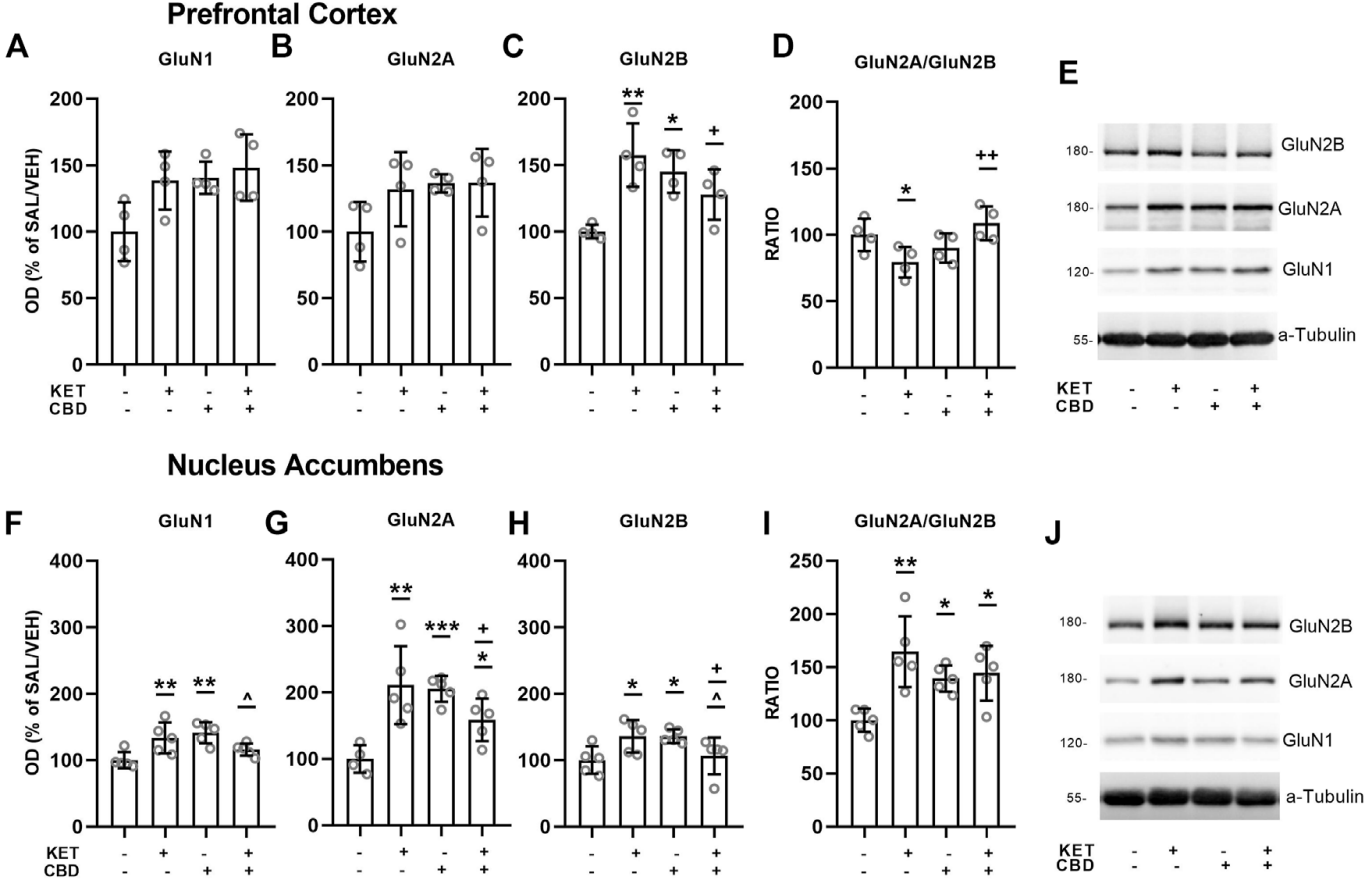
Effects of cannabidiol (CBD) treatment on ketamine (KET)-induced alterations of NMDA receptor subunit protein expression levels in the prefrontal cortex **(top row)** and the nucleus accumbens **(bottom row)**. **(A,F)**:GluN1; **(B,G)**: GluN2A; **(C,H)**: GluN2B; **(D,I)**: GluN2A/GluN2B ratio; **(E,J)**: A representative band of the protein of interest and the corresponding loading marker band. Data are presented as percentage of the SAL/VEH group. The optical density (OD) of each band was divided by the corresponding loading marker. Statistical analysis was performed by two-way ANOVA (Bonferroni’s post hoc test). *p < 0.05, **p < 0.01, ***p < 0.001 vs SAL/VEH; + *p*< 0.05, ++ *p*< 0.01 vs KET/VEH; ^ *p* < 0.05 vs SAL/CBD (*n* = 4 per group in the prefrontal cortex and *n* = 5 per group in the nucleus accumbens).

**FIGURE 4:**
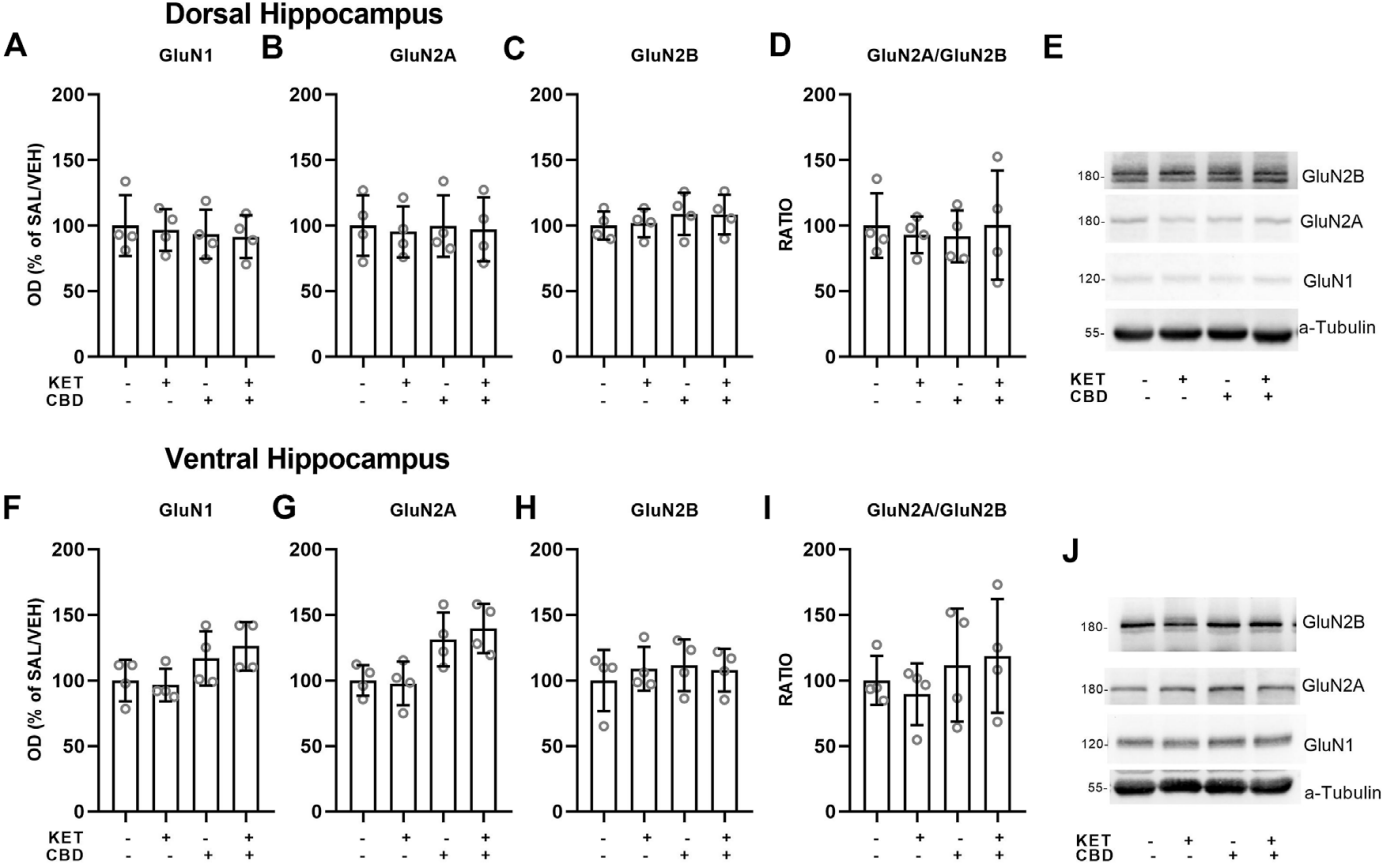
Effects of cannabidiol (CBD) treatment on ketamine (KET)-induced alterations of NMDA receptor subunit protein expression levels in the dorsal hippocampus **(top row)** and the ventral hippocampus **(bottom row)**. **(A,F)**: GluN1; **(B,G)**: GluN2A; **(C,H)**: GluN2B; **(D,I)**: GluN2A/GluN2B ratio; **(E,J)**: A representative band of the protein of interest and the corresponding loading marker band. Data are presented as percentage of the SAL/VEH group. The optical density (OD) of each band was divided by the corresponding loading marker. Statistical analysis was performed by two-way ANOVA.

Effects of CBD on KET-induced NMDA receptor subunit composition are presented in Figures 3, 4.

### Prefrontal Cortex

Two-way ANOVA for PFC GluN1 levels revealed a main effect of KET treatment [F_(1,12)_ = 4.89, *p* = 0.047] and a main effect of CBD treatment [F_(1,12)_ = 5.85, *p* = 0.032], without a statistically significant KET × CBD interaction (Figure 3A).

Regarding GluN2A subunit expression, no significant main effects or interactions were observed (Figure 3B).

Two-way ANOVA for PFC GluN2B protein expression levels showed a KET × CBD interaction [F_(1,12)_ = 14.71, *p* = 0.002]. GluN2B levels were significantly higher in KET/VEH (*p* = 0.002) and SAL/CBD (*p* = 0.019) rats, compared to SAL/VEH. Furthermore, GluN2B levels were significantly lower in KET/CBD rats, compared to KET/VEH (*p* = 0.034) (Figure 3C).

Two-way ANOVA for PFC GluN2A/GluN2B ratio showed a significant KET × CBD interaction [F_(1,12)_ = 10.90, *p* = 0.006]. Subsequent post hoc comparisons showed that the ratio was significantly lower in KET/VEH rats, compared to SAL/VEH (*p* = 0.031) and to KET/CBD (*p* = 0.005) (Figure 3D).

### Nucleus Accumbens

Two-way ANOVA for NAc GluN1 subunit protein expression levels showed a KET × CBD interaction [F_(1,15)_ = 14.30, *p* = 0.002]. NAc GluN1 levels were significantly higher in KET/VEH (*p* = 0.005) and SAL/CBD (*p* = 0.001) groups, compared to SAL/VEH, an effect that was not observed for KET/CBD group. Furthermore, NAc GluN1 levels were lower in KET/CBD rats, compared to SAL/CBD (*p* = 0.024) (Figure 3F).

Two-way ANOVA for NAc GluN2A subunit protein expression levels showed a KET × CBD interaction [F_(1,15)_ = 21.16, *p* < 0.001]. NAc GluN2A levels were significantly higher in KET/VEH (*p* = 0.001), SAL/CBD (*p* < 0.001), and KET/CBD (*p* = 0.032) groups, compared to SAL/VEH. Moreover, GluN2A levels were lower in KET/CBD group compared to KET/VEH group (*p* = 0.042) (Figure 3G).

Two-way ANOVA for NAc GluN2B subunit protein expression levels showed a KET × CBD interaction [F_(1,15)_ = 14.37, *p* = 0.002]. NAc GluN2B levels were significantly higher in KET/VEH and SAL/CBD groups, compared to SAL/VEH (*p* = 0.019; *p* = 0.019, respectively). Moreover, GluN2B levels were lower in KET/CBD group compared to SAL/CBD and KET/VEH group (*p* = 0.047; *p* = 0.048, respectively) (Figure 3H).

Two-way ANOVA for NAcGluN2A/GluN2B ratio showed a KET × CBD interaction [F_(1,15)_ = 7.74, *p* = 0.014]. NAc GluN2A/GluN2B ratio was significantly higher in KET/VEH (*p* = 0.001), SAL/CBD (*p* = 0.023) and KET/CBD group (*p* = 0.012), compared to SAL/VEH (Figure 3I).

### Dorsal and Ventral Hippocampus

No effects on NMDA receptor subunit protein expression levels were observed in the dorsal or ventral hippocampus (Figure 4).

CBD treatment reversed KET-induced effects on AMPA receptor subunit expression, in specific rat brain regions.

**FIGURE 5:**
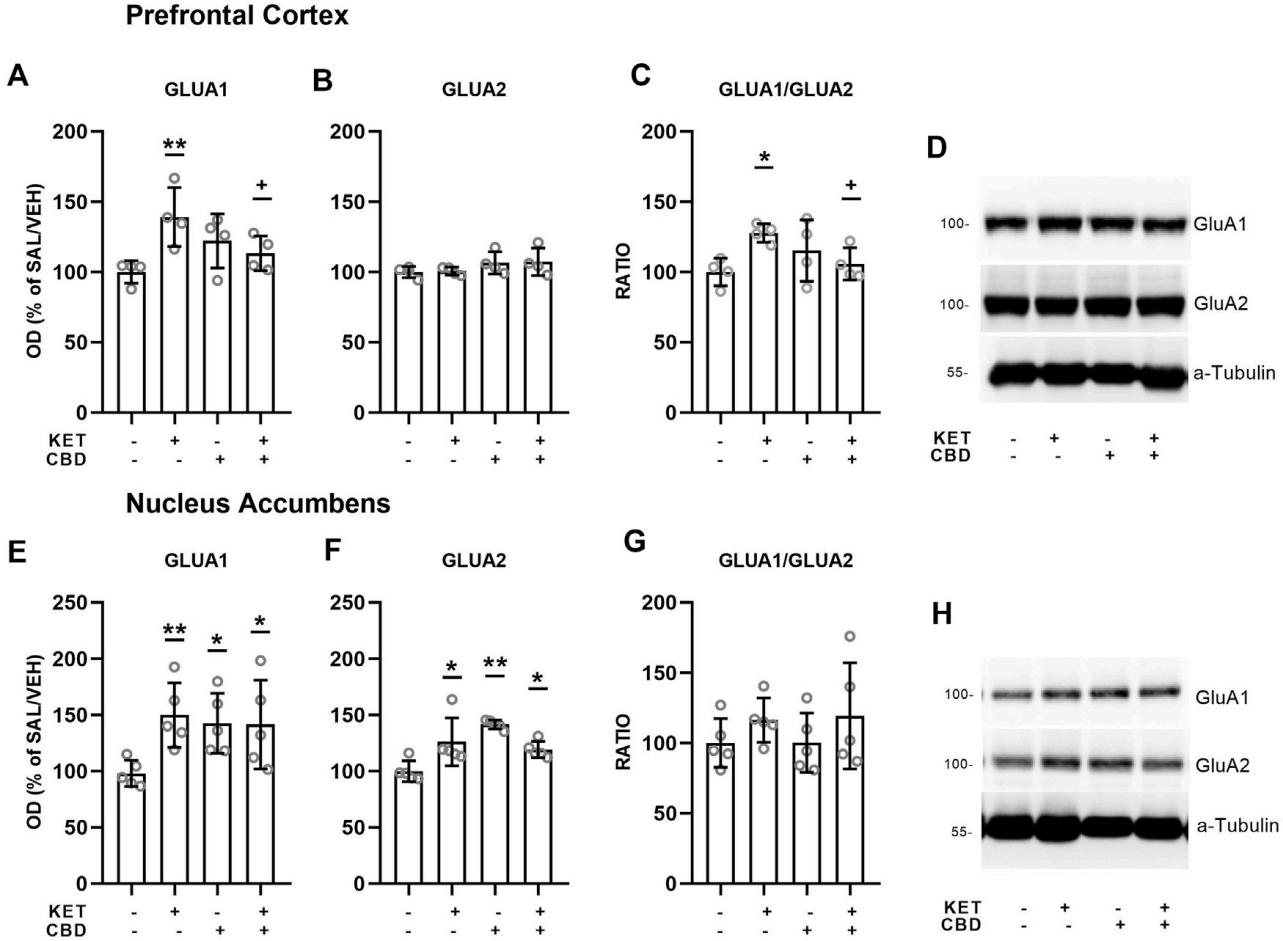
Effects of cannabidiol (CBD) treatment on ketamine (KET)-induced alterations of AMPA receptor subunit protein expression levels in the prefrontal cortex **(top row)** and the nucleus accumbens **(bottom row)**. **(A,E)**: GLUA1; **(B,F)**: GLUA2; **(C,G)**: GLUA1/GLUA2 ratio; **(D,H)**: a representative band of the protein of interest and the corresponding loading marker band. Data are presented as percentage of the SAL/VEH group. The optical density (OD) of each band was divided by the corresponding loading marker. Statistical analysis was performed by two-way ANOVA (Bonferroni’s post hoc test).*p < 0.05, **p < 0.01vs SAL/VEH; + *p*< 0.05 vs KET/VEH (*n* = 4 per group).

**FIGURE 6:**
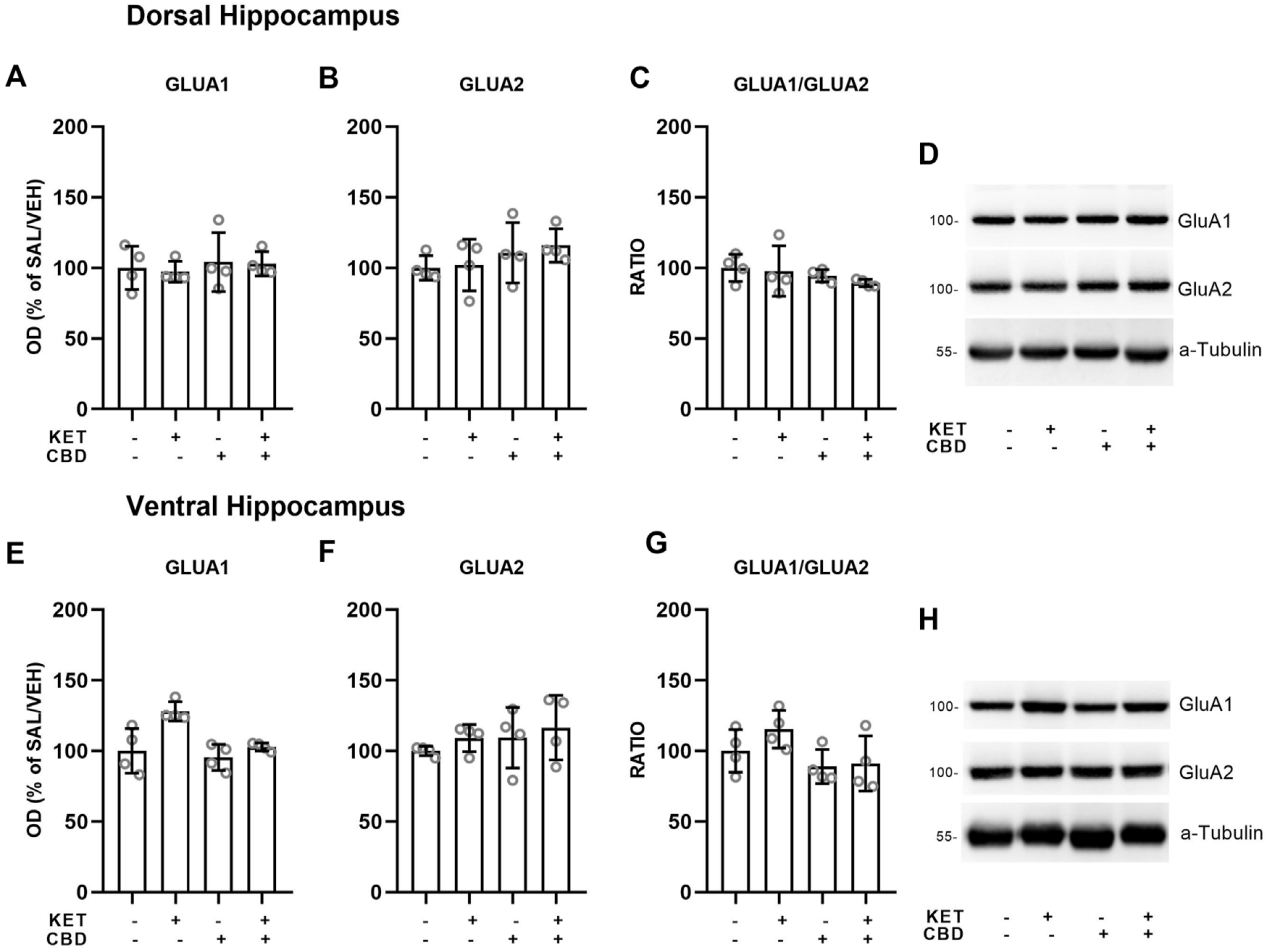
Effects of cannabidiol (CBD) treatment on ketamine (KET)-induced alternations of AMPA receptor subunit protein expression levels in the dorsal hippocampus **(top row)** and the ventral hippocampus **(bottom row)**. **(A,E)**: GLUA1; **(B,F)**: GLUA2; **(C,G)**: GLUA1/GLUA2 ratio; **(D,H)**: a representative band of the protein of interest and the corresponding loading marker band. Data are presented as percentage of the SAL/VEH group. The optical density (OD) of each band was divided by the corresponding loading marker. Statistical analysis was performed by two-way ANOVA.

Effects of CBD on KET-induced AMPA receptor subunit composition are presented in Figures 5, 6.

### Prefrontal Cortex

In the PFC, two-way ANOVAs showed KET × CBD interactions for the GluA1 subunit protein expression levels [F_(1,11)_ = 7.40, *p* = 0.020] and for the GluA1/GluA2 ratio [F_(1,11)_ = 5.89, *p* = 0.034]. GluA1 levels were higher in KET/VEH, compared to SAL/VEH (*p* = 0.005) and to CBD/KET (*p* = 0.042) (Figure 5A). The GluA1/GluA2 ratio was significantly higher in KET/VEH rats, compared to SAL/VEH (*p* = 0.034), and KET/CBD rats (*p* = 0.036) (Figure 5C).

### Nucleus Accumbens

In the NAc, two-way ANOVAs showed KET × CBD interactions for GluA1 [F_(1,11)_ = 4.43, *p* = 0.049] and GluA2 [F_(1,16)_ = 19.16, *p* < 0.001] subunit protein expression levels. GluA1 levels were significantly higher in KET/VEH (*p* = 0.011), SAL/CBD (*p* = 0.025), and CBD/KET (*p* = 0.028) rats, compared to SAL/VEH (Figure 5E). NAc GluA2 levels were significantly higher in KET/VEH (*p* = 0.004), SAL/CBD (*p* = 0.001), and CBD/KET (*p* = 0.026), compared to SAL/VEH (Figure 5F). The two-way ANOVA did not reveal any statistically significant effect for GluA1/GluA2 ratio.

### Hippocampus

In the Ventral Hippocampus, two-way ANOVAs showed main effects of KET [F_(1,12)_ = 12.73, *p* = 0.004] and CBD [F_(1,12)_ = 9.04, *p* = 0.01] on GluA1 subunit protein expression levels and a main effect of CBD on GluA2 levels [F_(1,12)_ = 6.35, *p* = 0.027], but no statistically significant KET × CBD interaction (Figure 6).

No effects on AMPA subunit levels were observed in the Dorsal Hippocampus (Figure 6).

CBD treatment reversed KET-induced ERK1/2 phosphorylation state effects in specific rat brain regions.

**FIGURE 7:**
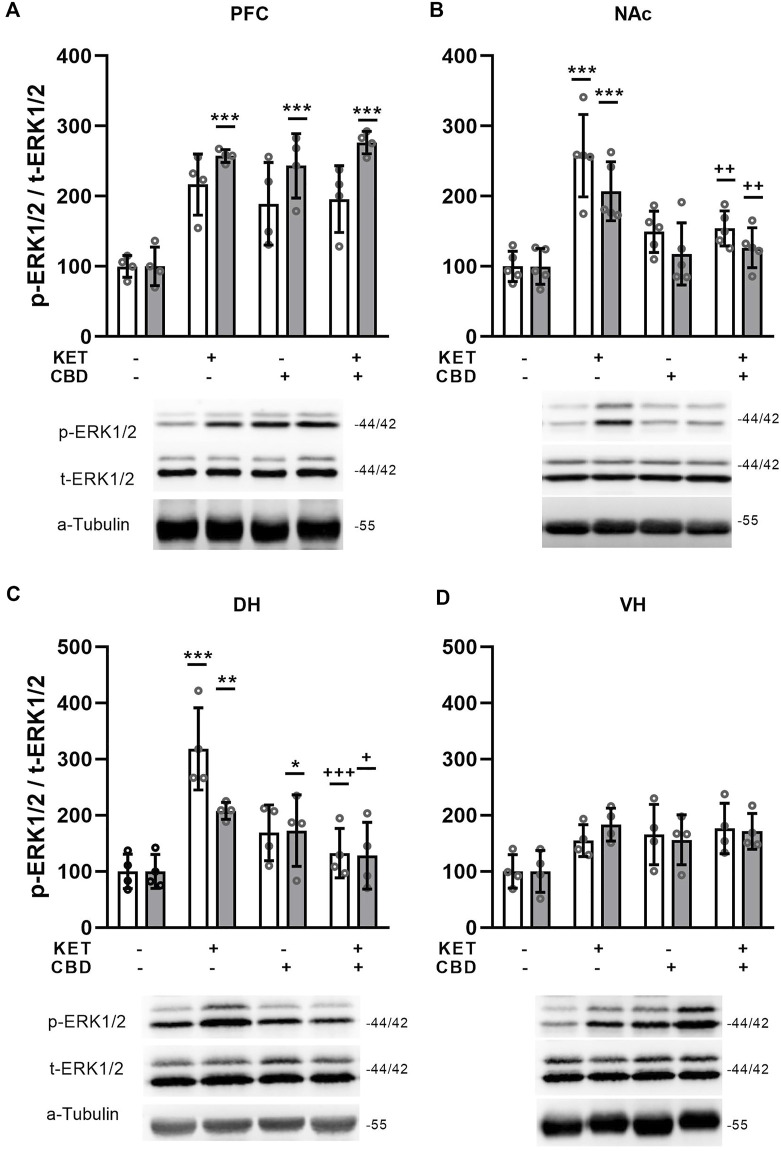
Effects of cannabidiol (CBD) treatement on ketamine (KET)-induced alterations in the p-ERK1/2 / total ERK1/2 ratio in **(A)** the prefrontal cortex, PFC; **(B)** the nucleus accumbens, NAc; **(C)** the dorsal hippocampus, DH; **(D)** the ventral hippocampus, VH. White bars: p-ERK1, t-ERK1 isoforms; gray bars: p-ERK2, t-ERK2 isoforms. Data are presented as percentage of the SAL/VEH group. The optical density (OD) of each band was divided by the corresponding loading marker. Statistical analysis was performed by two-way ANOVA (Bonferroni’s post hoc test).*p < 0.05, **p < 0.01, ***p < 0.001 vs SAL/VEH; + *p*< 0.05, ++ *p* < 0.01, +++ *p*< 0.001 vs KET/VEH (*n* = 5 per group in the nucleus accumbens and *n* = 4 per group in all other regions).

Effects of CBD on KET-induced ERK1/2 phosphorylation state are presented in Figure 7 **.**

### Prefrontal Cortex

Two way ANOVA showed a KET × CBD interaction for p-ERK2/t-ERK2 ratio [F_(1,12)_ = 19.29, *p* = 0.001]. Τhe p-ERK2/t-ERK2 ratio was significantly higher in KET/VEH, SAL/CBD, and KET/CBD, compared to SAL/VEH (*p* < 0.001; *p* < 0.001; *p* < 0.001, respectively) (Figure 7A).

### Nucleus Accumbens

In the NAc, two-way ANOVAs showed KET × CBD interactions for p-ERK1/t-ERK1 and p-ERK2/t-ERK2 ratios [F_(1,16)_ = 13.63, *p* = 0.002; F_(1,16)_ = 9.30, *p* = 0.008, respectively]. Subsequent post-hoc comparisons showed that the p-ERK1/t-ERK1 ratio was significantly higher in KET/VEH rats, compared to SAL/VEH (*p* < 0.001) and KET/CBD rats (*p* = 0.003). Similarly, KET alone increased the p-ERK2/t-ERK2 ratio, compared to SAL/VEH (*p* < 0.001) and KET/CBD rats (*p* = 0.003) (Figure 7B).

### Hippocampus

In the Dorsal Hippocampus, KET × CBD interactions were observed for the p-ERK1/t-ERK1 [F_(1,12)_ = 24.31, *p* < 0.001] and the p-ERK2/t-ERK2 [F_(1,12)_ = 10.68, *p* = 0.007] ratios. Subsequent post-hoc comparisons showed that in KET/VEH rats the p-ERK1/t-ERK1 ratio was significantly higher compared to SAL/VEH (*p* < 0.001) and KET/CBD (*p* < 0.001). Furthermore, the p-ERK2/t-ERK2 ratio was significantly higher in KET/VEH compared to SAL/VEH (*p* = 0.007) and KET/CBD (*p* = 0.032), and significantly higher in SAL/CBD rats, compared to SAL/VEH (*p* = 0.048) (Figure 7C).

No effects in ERK1/2 phosphorylation state were observed in the ventral hippocampus (Figure 7D).

## Discussion

The goal of the present study was to evaluate neuroplastic changes induced by repeated KET administration, which is used as an experimental model of schizophrenia, and to assess the modulatory role of CBD treatment, which would indicate its antipsychotic potential.

The present findings have shown a robust increase in spontaneous and habituated motor activity in KET-treated rats following a 10-day period of chronic administration at the sub-anesthetic dose of 30 mg/kg (i.p.). These behavioral changes were reversed to normal by subsequent chronic CBD treatment. Concerning the expression of glutamate receptors, the current findings have shown regionally dependent KET-induced constitutional alterations in NMDA and AMPA receptors that were modified by subsequent CBD treatment. Last but not least, chronic KET administration induced an increase in ERK1/2 phosphorylation state in all regions examined, apart from the ventral hippocampus that was modulated by subsequent CBD treatment.

Spontaneous and habituated motor activity were found to be increased following chronic KET administration, while this behavioral stimulation was reduced by CBD treatment. Notably, the reactivity of KET-treated rats was achieved following the 10-days administration schedule at the dose of 30 mg/kg. The 7-days KET administration schedule failed to induce hyperlocomotion, which points out that the three additional daily doses are crucial for establishing a positive-like symptomatology.

Towards the same direction, a number of studies has shown that 5 daily doses of 30 mg/kg KET administration were not sufficient to produce hyperactivity (Becker et al., 2003, 2009; Schumacher et al., 2016), even if this 5-days administration pattern induced a hyper-responsivity to psychostimulants (Becker et al., 2003; Schumacher et al*.*, 2016). It should be noted that longer duration of KET administration or even higher doses did not induce any alteration in motor activity (Schumacher et al., 2016; Luo et al., 2020). This deviation among the latter studies and our findings could be attributed to differences in the experimental design, including the amount of KET dosing and the experimental animal used. Based on our findings and previous studies, it could be suggested that a 10-days KET administration at the dose of 30 mg/kg is a critical experimental procedure/timeline ensuring enhanced spontaneous and habituated motor activity, while shorter duration of the administration period or higher KET dosing are not appropriate. This hyperlocomotion is a manifestation of impairments in exploration, reactivity, and behavioral stimulation, that reflects positive symptomatology of schizophrenia-like disorders (Powell and Miyakawa, 2006). Interestingly, our results have shown that CBD attenuated ketamine-induced hyperlocomotion, in line with the attenuating role of CBD on amphetamine-induced locomotor sensitization that models schizophrenia (Renard et al., 2016). It is important to mention that in this study CBD was administered locally in the NAc and, thus, our results provide novel information concerning the systemic administration of CBD using another pharmacological model for mirroring schizophrenia. Concerning NMDA receptor subunit expression in the PFC, our results have shown that 10-days KET administration led to increased GluN2B subunit protein expression and to decreased GluN2A/GluN2B ratio. Interestingly, these effects were reversed by subsequent CBD administration. In the NAc, GluN1, GluN2A, and GluN2B subunit protein expression increased after repeated KET administration and the GluN2A/GluN2B ratio also increased. CBD treatment attenuated GluN1 and GluN2A levels and reversed GluN2B protein expression. Our findings have shown that repeated KET administration induced an opposite effect concerning the GluN2A/GluN2B ratio, since GluN2A levels increased most in the NAc, while GluN2B levels were higher in the PFC.

Hippocampal NMDA subunit composition was not affected by KET, which suggests that this specific pattern of repeated KET administration does not impact glutamate receptor composition in general, but it affects specific schizophrenia-associated brain regions. On the other hand, a recent study (Luo et al., 2020), has shown decreased hippocampal levels of all non-structural NMDA subunits after 10, 14, and 28 days of daily KET administration in mice, while GluN1 levels were reported intact. This lack of consistency among these results and our findings could be attributed to the different experimental protocol, species used and tissue dissection (dorsal—ventral divisions vs entire hippocampus).

Interestingly, the GluN2A/GluN2B ratio, which is thought to be increased in an activity-dependent manner in the synapses (Chen and Bear, 2007; Yashiro and Philpot, 2008; Xu et al., 2009), was found decreased in the PFC and increased in the NAc. Thus, the increased GluN2A/GluN2B ratio in the NAc along with the lower ratio in the PFC can be associated with a hyperactivity state in the NAc and hypoactivity in the PFC, exhibiting a valuable physiological association with schizophrenia pathophysiology. Interestingly, CBD normalized the ratio in the PFC, while it failed to reverse it in the NAc. This finding suggests a neurobiological correlate that could potentially explain several findings on the robust counteracting effect of CBD on cognitive and negative-like symptomatology (Gururajan et al., 2011; Gururajan et al., 2012; Osborne et al., 2017; Kozela et al., 2020; Rodrigues da Silva et al., 2020), along with its questionable ability to counteract positive-like deficits (Long et al., 2010; Valvassori et al., 2011; Brakatselos et al., 2021). Moreover, it is also important to note that GluN2A/GluN2B ratio has been shown to control whether the synapses undergo LTD. or LTP (Yashiro and Philpot, 2008; Xu et al., 2009). This notion supports the importance of this ratio for neuroplastic and subsequent neuroplastic processes and highlights the complex effects of KET on brain neuroplasticity.

Overall, our data show a repeated KET-induced increase in GluN1, GluN2A, and GluN2B subunits in the PFC and the NAc. Taking into consideration previous studies that support the dampening effects of sub-anesthetic KET on these subunits’ protein expression (Brakatselos et al., 2021; Piva et al., 2021), we could postulate that the increase observed in the current study can be related with homeostatic, compensatory mechanisms that function towards the normalization of neurotransmission and neuroplastic processes after sustained KET exposure. In this case, CBD treatment in a crucial time frame before the protein expression read-out, modulates KET-induced changes only in the PFC, thus exhibiting a region-specific homeostatic role.

Concerning AMPA receptor protein expression in the PFC, an increase in GluA1 subunit expression was observed, which resulted in subsequent increase of the GluA1/GluA2 ratio, both attenuated by CBD treatment. In the NAc, an increase of AMPA receptor subunit levels was observed following KET administration, an effect that was not modified by CBD treatment. The present findings indicate that repeated 10-days repeated KET induced an over-expression in AMPA receptor subunits in brain regions related to the neurobiology of schizophrenia, which was normalized by CBD treatment only in the PFC. These AMPA related effects are in parallel to GluN1, GluN2A, and GluN2B over-expression and point out the region-dependent neuroplastic changes induced by repeated KET administration that were modulated by CBD treatment.

Taking together our data concerning chronic KET effects on glutamate receptor protein expression, it is clear that the main regions affected were the PFC and the NAc, showing increased expression of most subunits studied, but displaying opposite GluN2A/GluN2B ratio. Our findings are in line with previous studies of sub-chronic or chronic treatment with ketamine and other non-competitive NMDA antagonists. Chronic ketamine treatment has been previously shown to increase the GluN1/PSD95 and the GluN2A/PSD95 ratio, which is expected to impact calcium signaling (Lisek et al., 2017). In addition, NMDA subunit protein expression increases after chronic MK801 (Rammes et al., 2001; Zhou et al., 2020) or PCP (Yu et al., 2002; Anastasio and Johnson, 2004) treatments, and this increase has been linked with hyperfunctional NMDA receptors (Yu et al., 2002). On the other hand, studies have also shown inhibitory (Ding et al., 2016; Liu et al., 2018; Sun et al., 2021) or no effects (Uttl et al., 2018) of these drugs on NMDA subunit expression, possible associated with differences in the dosage regimen.

Data arising from postmortem (patients with schizophrenia) studies are even more puzzling to be associated with our preclinical findings due to the complex status of the subjects studied in terms of disease stage, drug presence, patient compliance, and potential comorbidities. However, they also indicate that schizophrenia *per se*, or with co-existing medication affects NMDA and AMPA protein expression levels and associated neurobiological indices linked with neuroplastic indices (Kristiansen et al., 2006; Zavitsanou et al., 2008).

Cannabidiol counteracting -or no- effects, on the above-mentioned KET induced changes, alongside with its limited action on NMDA and AMPA receptor expression *per se* could not be easily attributed to a specific pharmacological target of CBD, rather than to its complex pharmacological profile. CBD has been shown to exert antipsychotic action through its action on 5-HT_1A_ receptors (Renard et al., 2016), it’s action on the endocannabinoid system (Leweke et al., 2012), or indirectly by modulating specific components of signal transduction pathways (Hudson et al., 2019). Additionally, a recent study showed for the first time that CBD acts as a negative allosteric modulator of AMPA receptors (when are constituted by GluA1/GluA2 subunits specifically) (Yu et al., 2020), which may also be associated with the present findings.

Based on our findings, CBD administration *per se* increased the expression of specific NMDA and AMPA receptor subunits in the PFC and NAc and the phospho-ERK2 in the prefrontal cortex. CBD attenuated the KET-induced effects although it induced specific effects in the same direction as compared to KET. This phenomenon could be attributed to the CBD activating role *per se* on specific glutamatergic components in the PFC and NAc that are associated with potential neuroprotective mechanisms. In support of these findings, CBD alone has been found to increase glutamate neurotransmission (Linge et al., 2016; O’Neill et al., 2021); an effect which is also observed following subanesthetic KET administration (Stone et al., 2012; Abdallah et al., 2018). Moreover, the antidepressant effect of CBD at similar doses used in our study has been associated with the elevation of neuroplasticity indices, including BDNF, potentially through NMDA and AMPA activation (Silote et al., 2019). A recent study has shown that CBD’s antidepressant effect relies on AMPA receptor activation (Sartim et al., 2021), supporting the abovementioned mechanistic explanation. Noteworthy, we have shown that repeated CBD administration did not act additively following repeated KET administration in this manner, but on the contrary, it depressed KET-induced effects in terms of NMDA subunit expression, which is associated with this schizophrenia-like profile. It could be postulated that CBD acts in a biphasic manner—under physiological conditions it promotes neuroplasticity, including the elevation of BDNF and related markers, while it modulates aberrant function of neuroplasticity indices when they are evoked under a pathophysiological state. Importantly, it should be noted that the protein expression alterations related to the glutamatergic system are not solely associated with the respective motor profiles induced by CBD, KET, or KET/CBD co-administration; an effect that needs further investigation.

ERK1/2 phosphorylation state was increased in all regions studied, except for VH, while this effect was counteracted by CBD treatment in the NAc and the DH. Interestingly, ERK1/2 phosphorylation does not follow the expression pattern of NMDA subunits in all brain regions. Moreover in our previous report, we showed that after acute KET administration, p-ERK1/2 levels decreased only in the NAc, while GluN2B levels decreased in both the NAc and the VH (Brakatselos et al., 2021). Based on our findings, it could be suggested that the chronic KET-induced increase in the glutamate receptor subunit expression could be linked to enhanced ERK1/2 signaling, and interestingly, CBD modulates this effect in a region-specific manner. Another study that examined ERK1/2 phosphorylation state (Luo et al., 2020), has reported reduced hippocampal total ERK1/2 and phospho ERK1/2 levels after a 28-days long treatment with KET. It should be noted that the ratio of p-ERK/t-ERK for both isoforms is probably unaffected by the treatment since total and phospho-ERK are reduced in the same degree. Taking into consideration that in the aforementioned study protein expression levels are analyzed in the entire hippocampus, it could be suggested that our findings are in agreement, as far as the VH ERK1/2 phosphorylation is concerned. Apart from our previous report mentioned above, Hudson et al., 2019 also showed that CBD *per se* does not affect ERK1/2 phosphorylation but attenuates acute THC-induced increase of p-ERK1/2, which is correlated with salience attribution, a behavioral index that is linked with negative symptomatology. These few reports supporting that CBD attenuates aberrant status of ERK1/2 phosphorylation which is related to schizophrenia-related symptomatology, converge with studies regarding antipsychotic drugs effect on p-ERK1/2. In particular it is shown that antipsychotics *per se* induce opposite effects of p-ERK state as compared to p-ERK alterations in psychosis or schizophrenia models (Ruso-Julve et al., 2019). These reports concerning antipsychotic action on p-ERK are in line with present findings concerning CBD effects.

This study has potential limitations. The behavioral experiments are focusing on motor activity parameters (ambulation). These motor variables in the open-field could be further enriched in order to expand our data with more detailed information. Finally, correlation analyses could be important so as to provide significant information on the issues related to the questions of the current manuscript.

## Conclusion

The present results show, for the first time, a stimulated motor output coupled with protein hyper-expression of NMDA and AMPA subunits following chronic KET administration that were attenuated by CBD treatment, in a region-dependent manner. These neuroplastic changes, including ERK1/2 activation, show a specific glutamatergic-related status that is affected by repeated KET administration that models schizophrenia. Moreover, CBD displays a profile of a potential antipsychotic drug, since it counteracts increased ambulatory activity and modulates the expression pattern of glutamatergic receptors and downstream signaling. The present findings contribute to the mapping of the complex and heterogeneous effects of CBD and provide novel information concerning its antipsychotic potential using a specific design of chronic KET administration, that contributes to psychosis and schizophrenia modeling.

## Data Availability

The raw data supporting the conclusion of this article will be made available by the authors, without undue reservation.
