# Supplementary material for: CBD Effects on Motor Profile and Neurobiological Indices Related to Glutamatergic Function Induced by Repeated Ketamine Pre-Administration
Source: Front Pharmacol. 2021 Oct 27;12:746935. doi: 10.3389/fphar.2021.746935 (PMC8578683; doi:10.3389/fphar.2021.746935)

Supplementary Material

Western Blot uncropped images of whole membrane

The images are presented as they were exported from ChemiDoc XRS software in the form of TIF file, without any processing in any kind of software, except rotation.

Prefrontal cortex-NMDA

1.PFC – NR2B

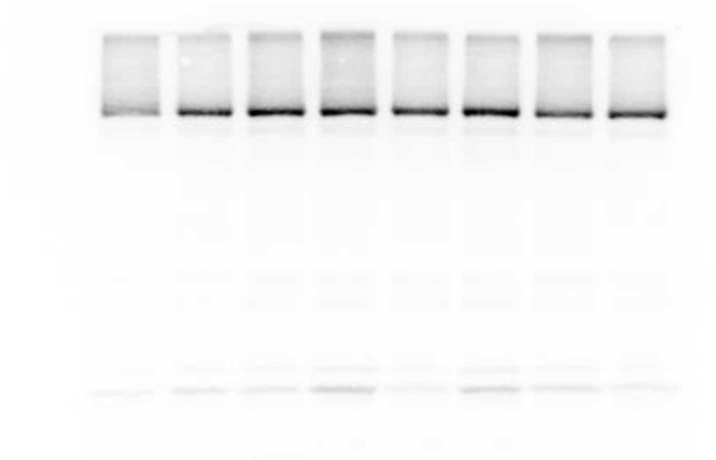

2.PFC-NR2A

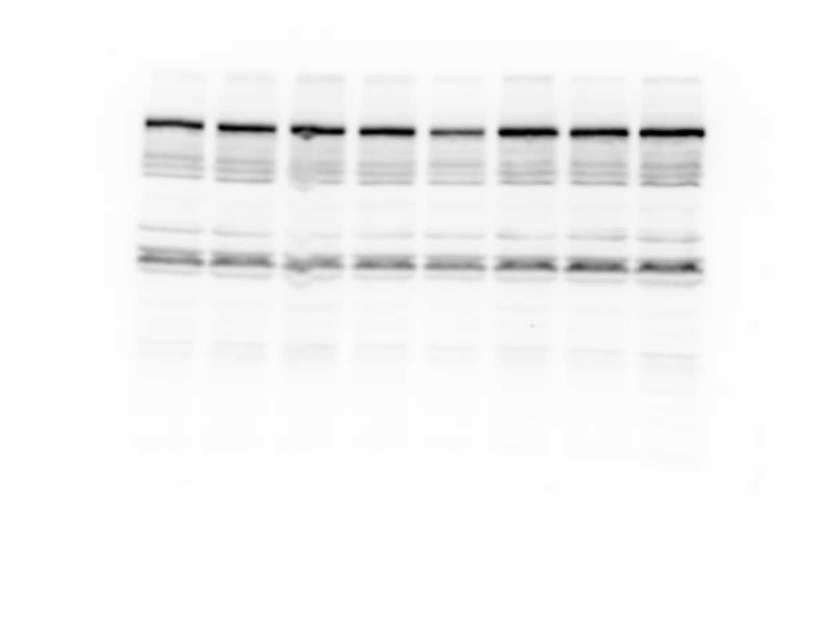

3.PFC – NR1

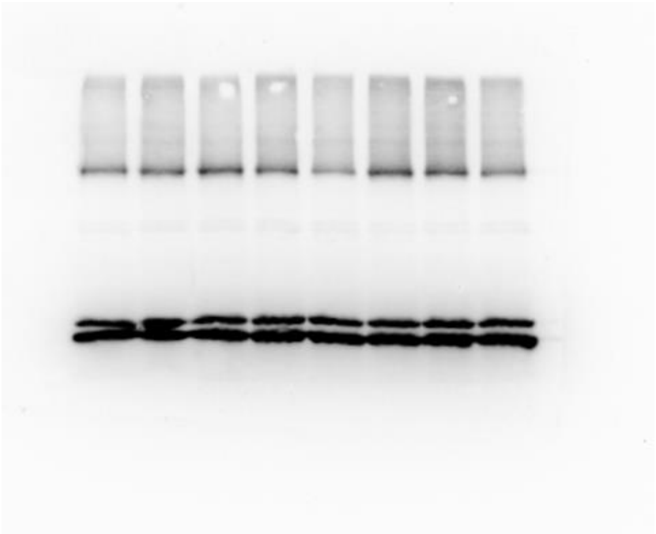

4. PFC – Tubulin

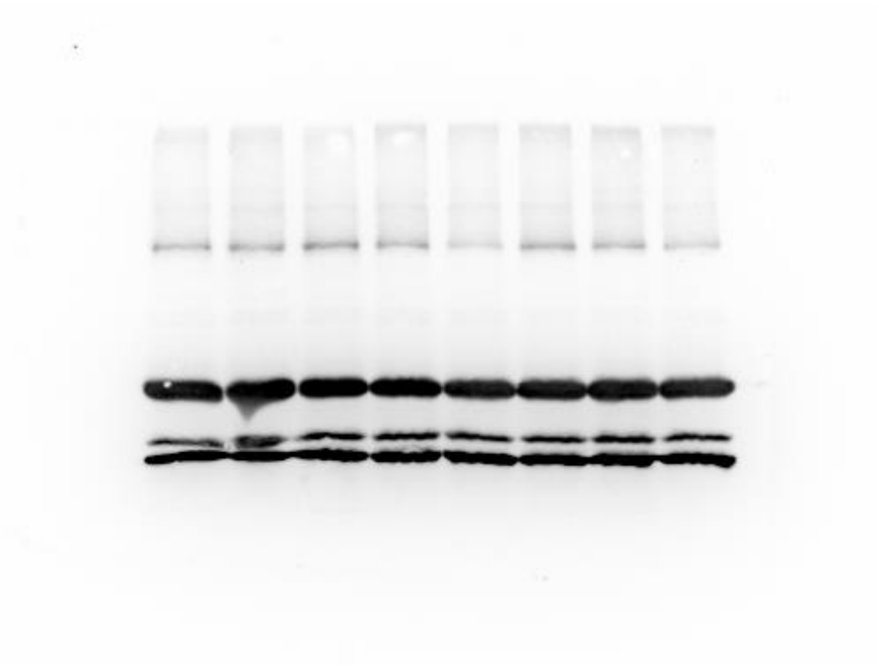

Nucleus accumbens – NMDA

1. NAc-NR2B

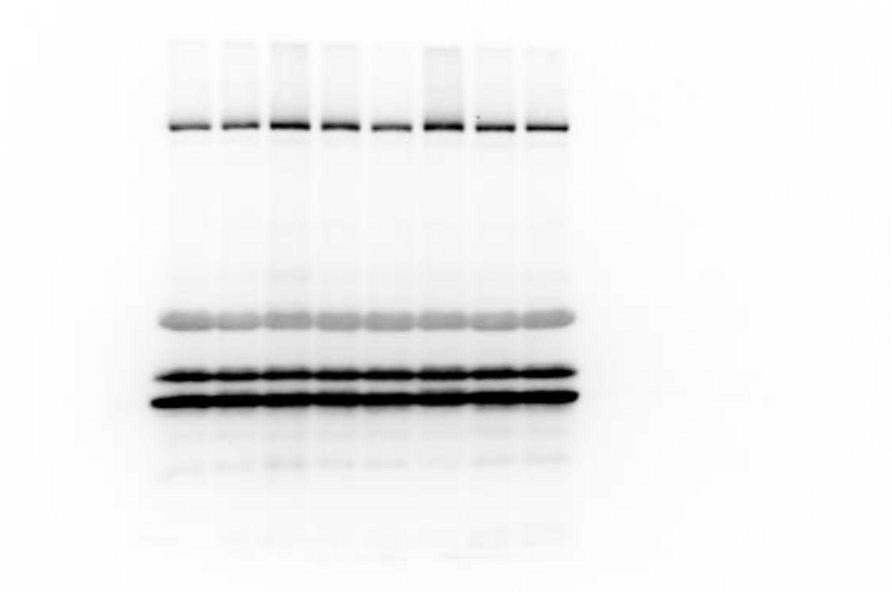

2. NAc-NR2A

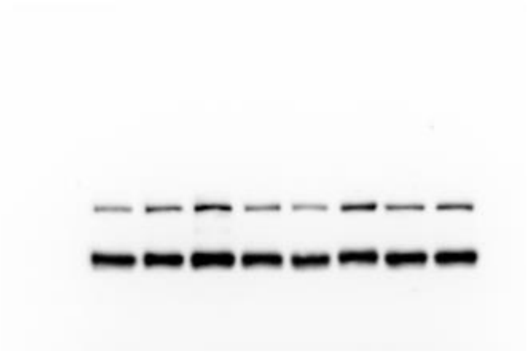

3. NAc-NR1

4.NAc-Tubulin

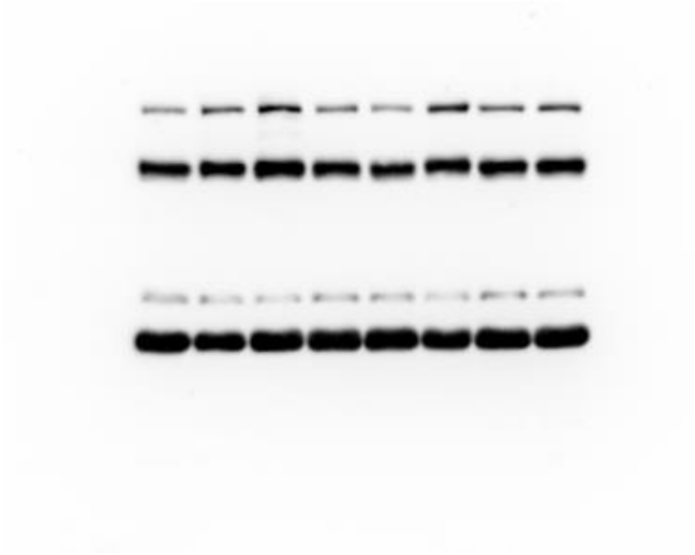

Ventral hippocampus – NMDA

1.VH-NR2B (Here we have the upper part of 2 gels transferred to one membrane)

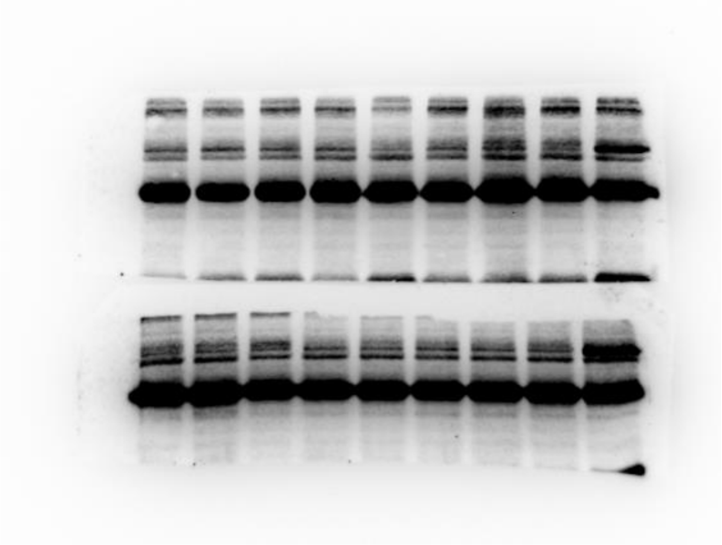

2. VH-NR2A

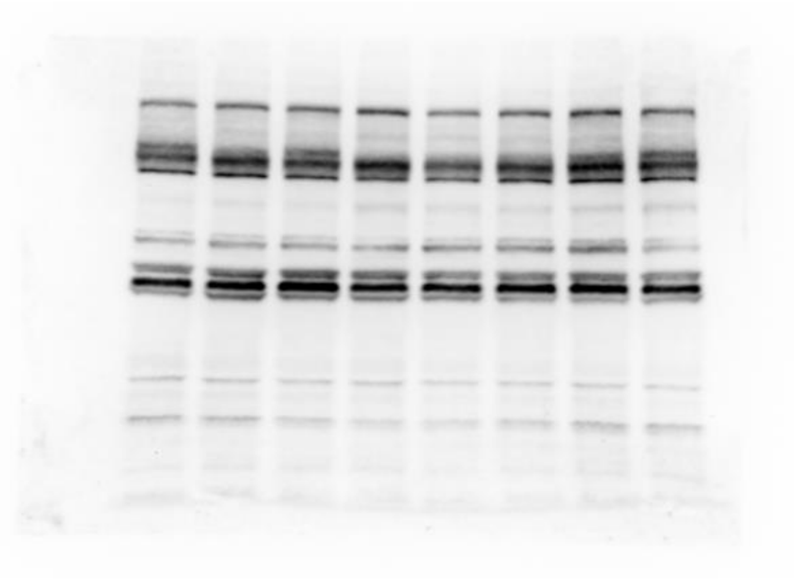

3.VH-NR1

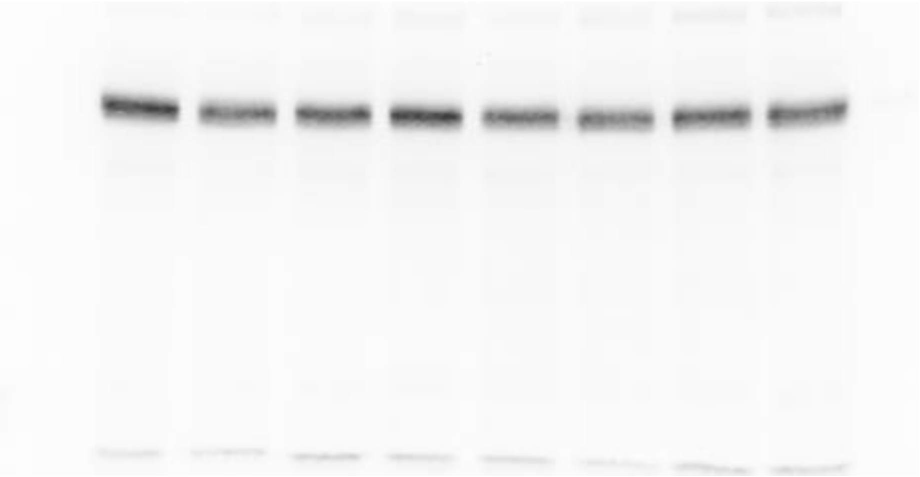

4. VH-tubulin

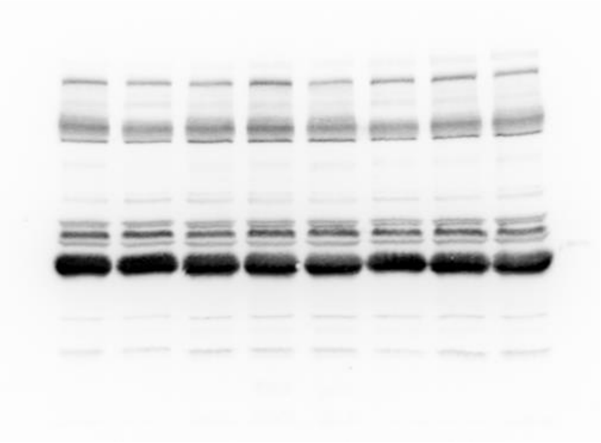

Dorsal hippocampus – NMDA

1. DH-NR2B

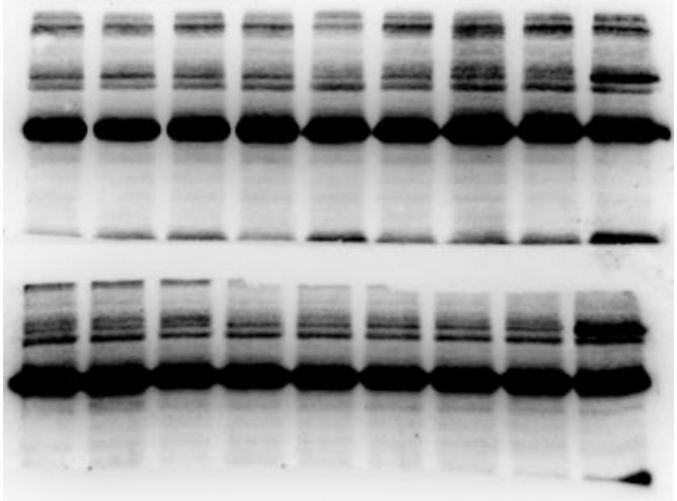

2.DH-NR2A

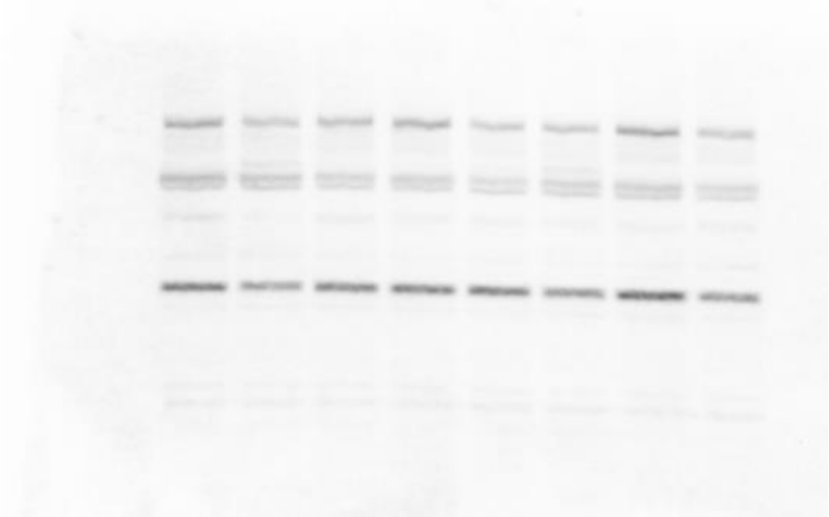

3.DH-NR1

4.DH-tubulin

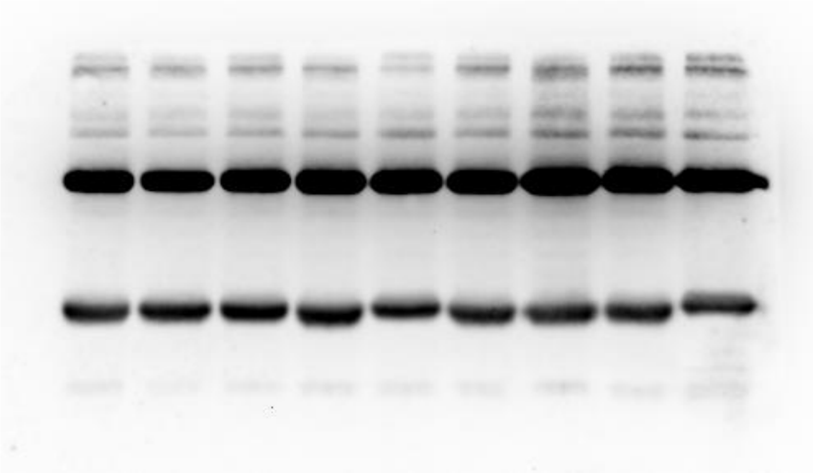

PFC – AMPA

1. PFC-GluA1

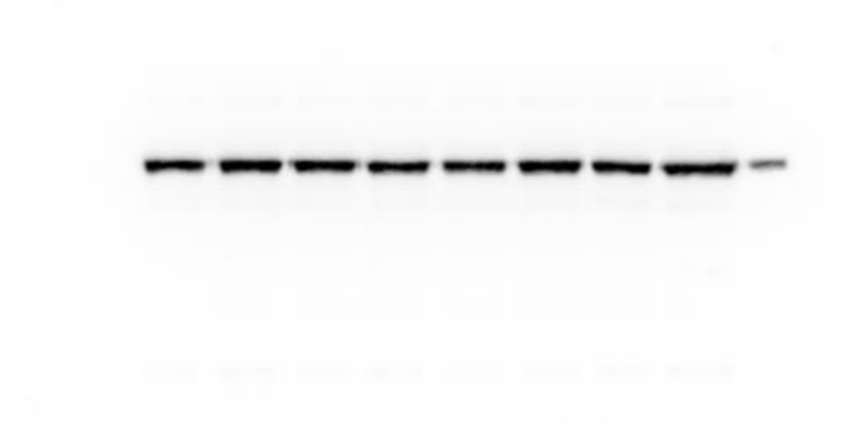

2. PFC-GluA2

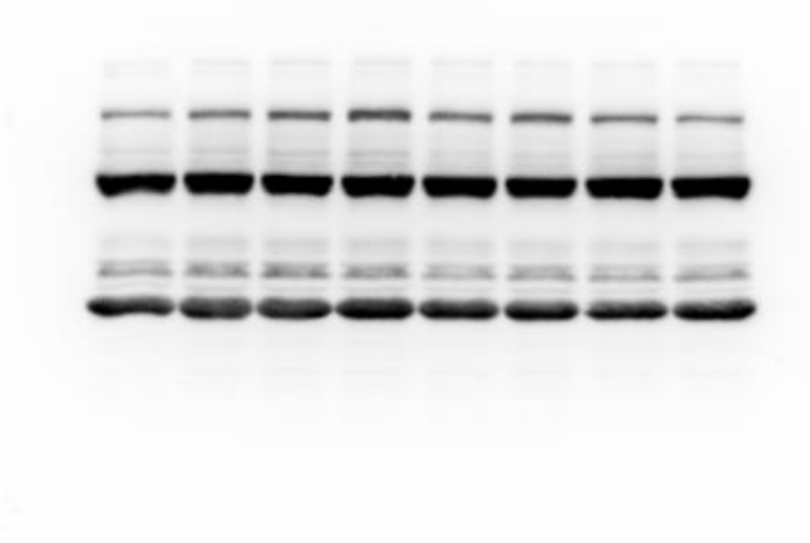

3.PFC-Tubulin

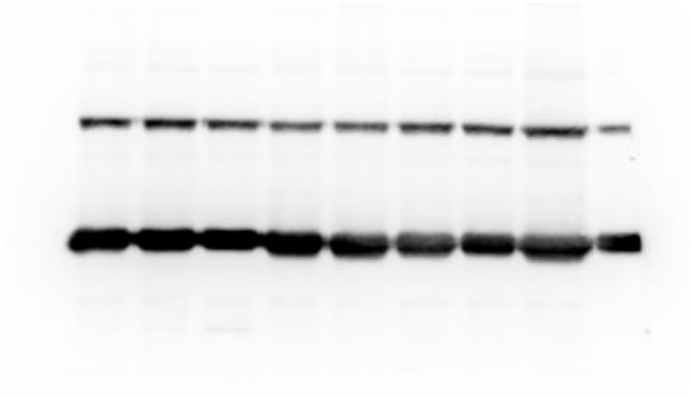

NAc – AMPA

1.NAc-GluA1

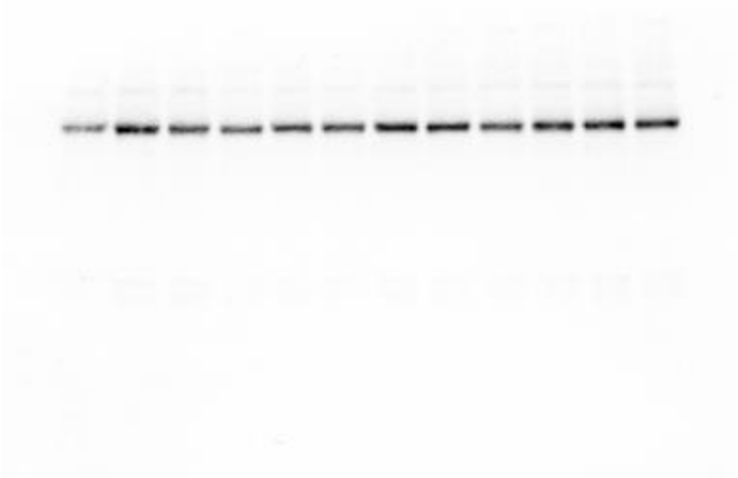

2.NAc-GluA2

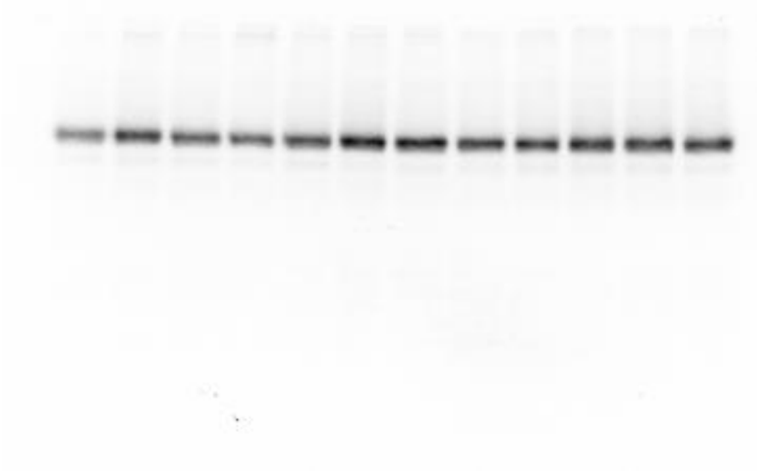

3.NAc-Tubulin

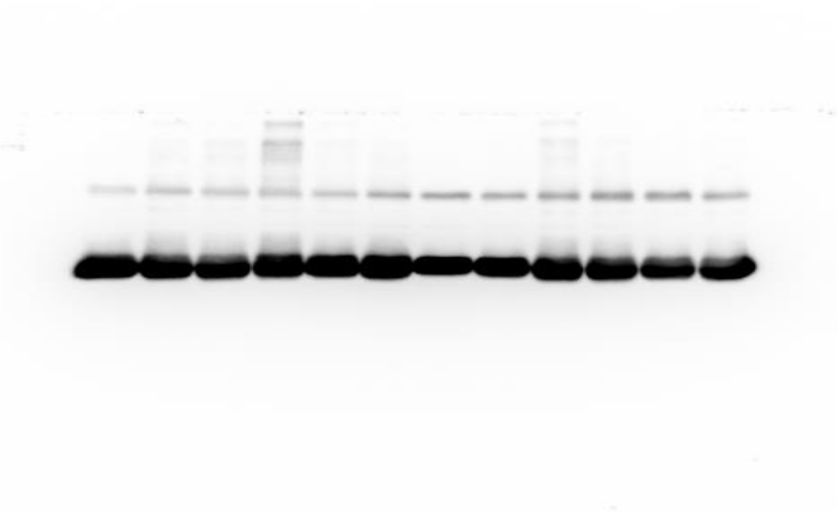

VH-AMPA

1. VH-GluA1

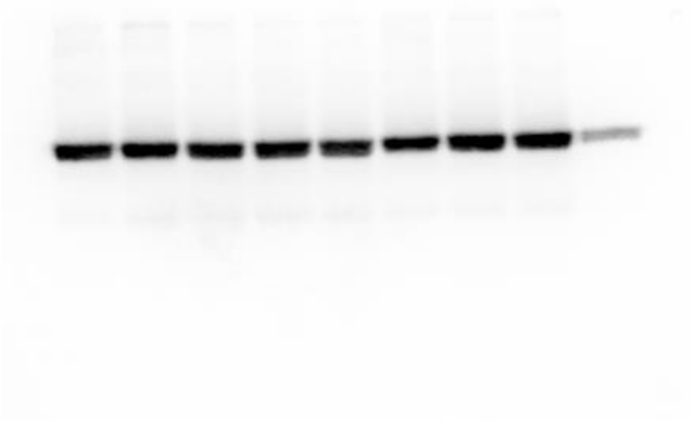

2. VH-GluA2

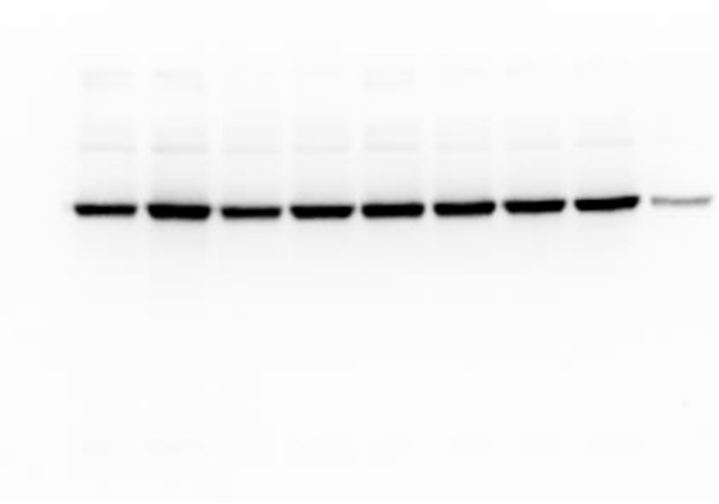

frontiers

3.VH – Tubulin

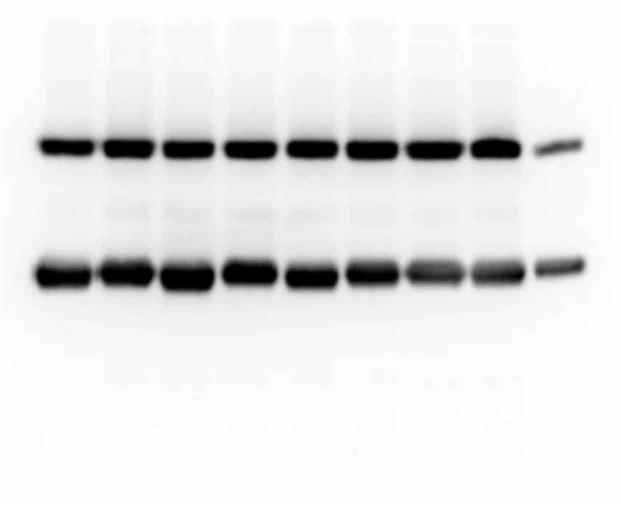

DH-AMPA

1.DH-GlA1

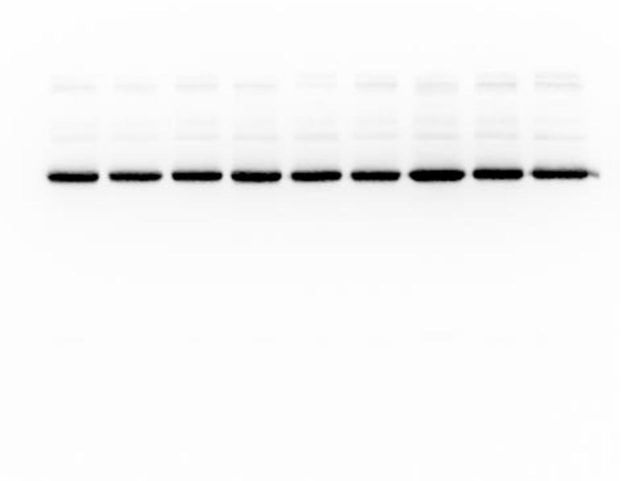

2.DH-GluA2

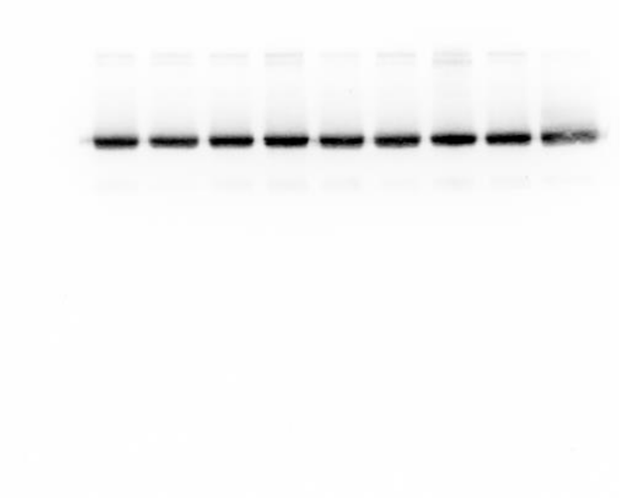

3.DH-Tubulin

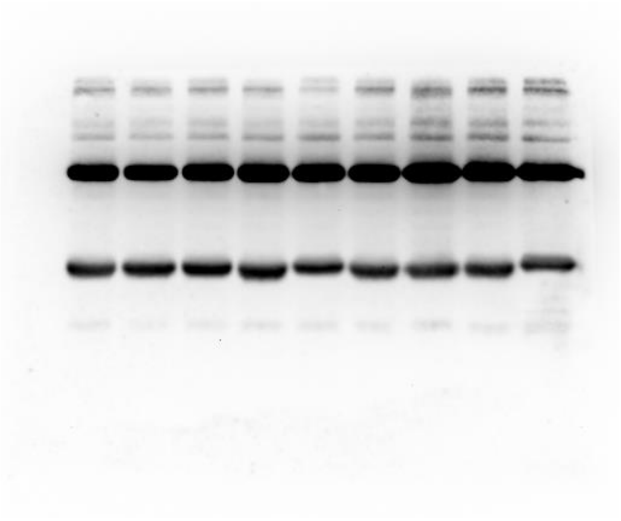

PFC – ERK1/2

1.PFC – Phospho ERK1/2

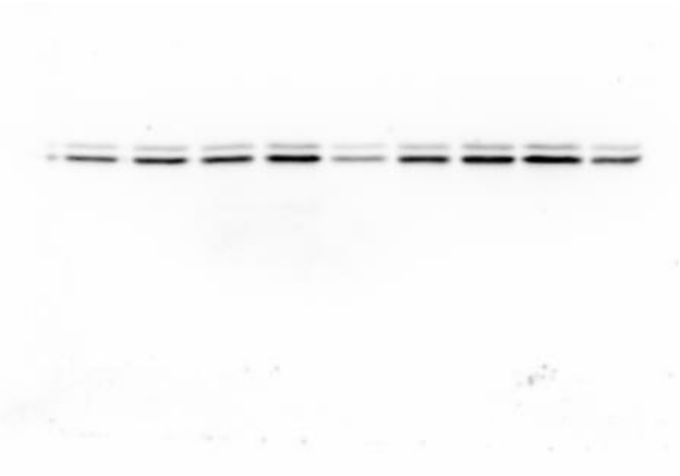

2.PFC – total ERK1/2

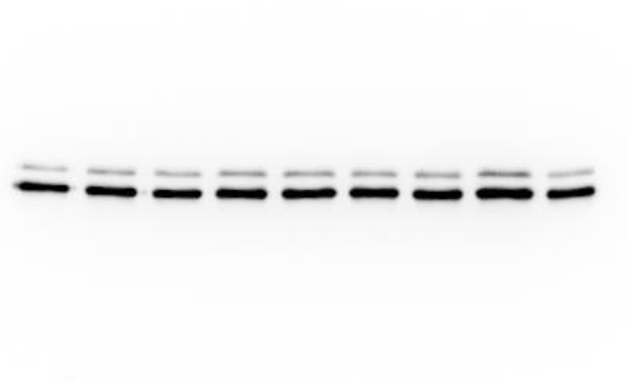

3.PFC – Tubulin

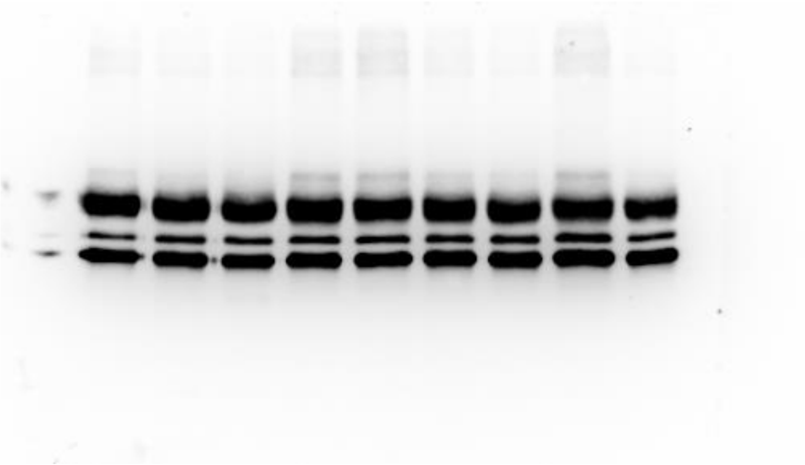

NAC – ERK1/2

1.NAc – Phosppho ERK1/2

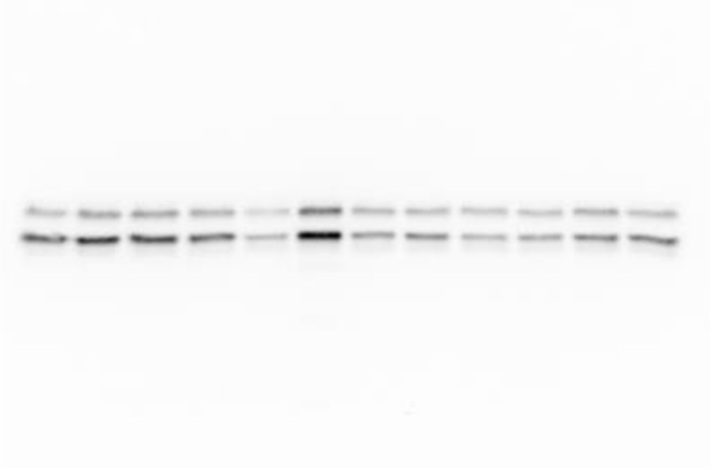

2.NAc – total ERK1/2

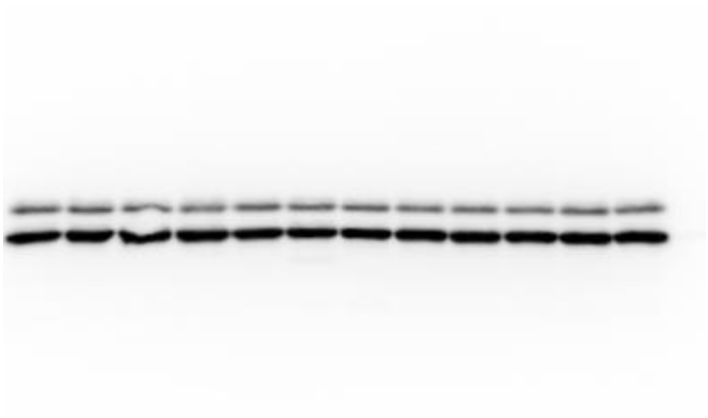

3.NAc – Tubulin

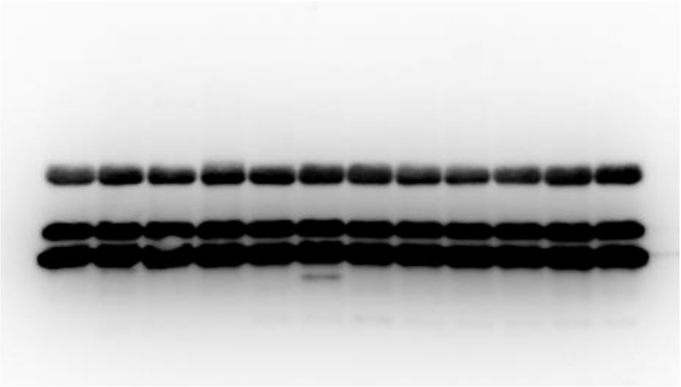

VH – ERK1/2

1.VH – Phosppho ERK1/2

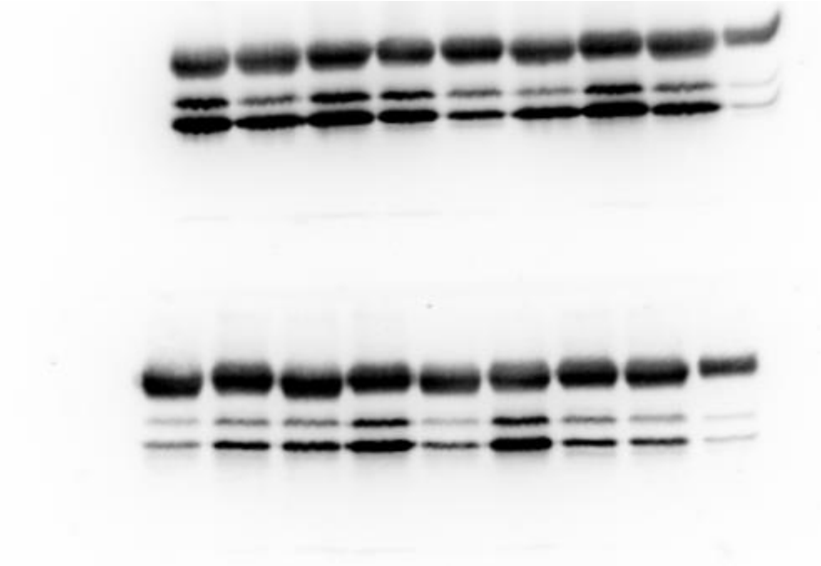

2.VH – total ERK1/2

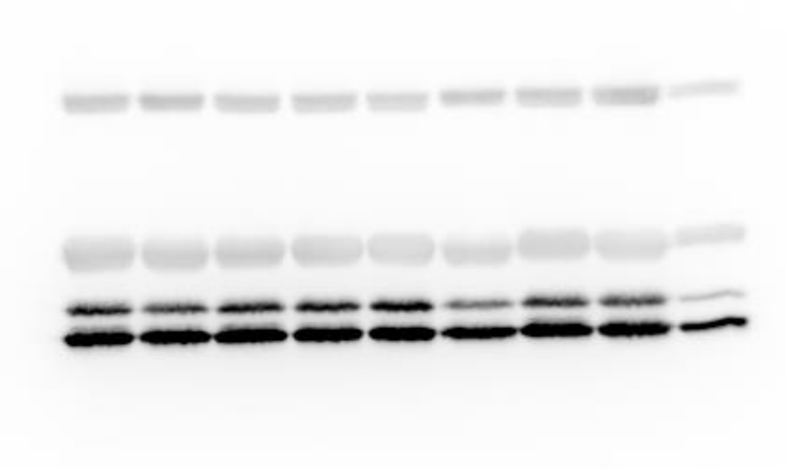

3.VH – Tubulin

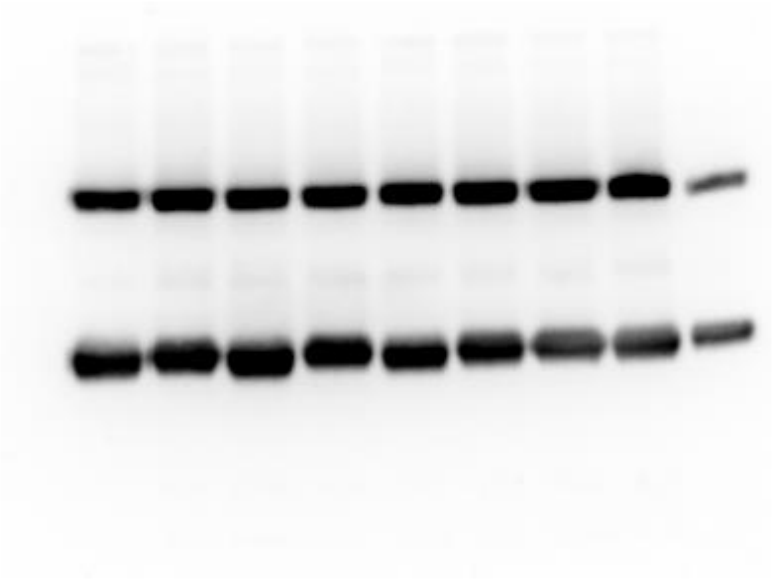

DH – ERK1/2

1.DH – Phospho ERK1/2

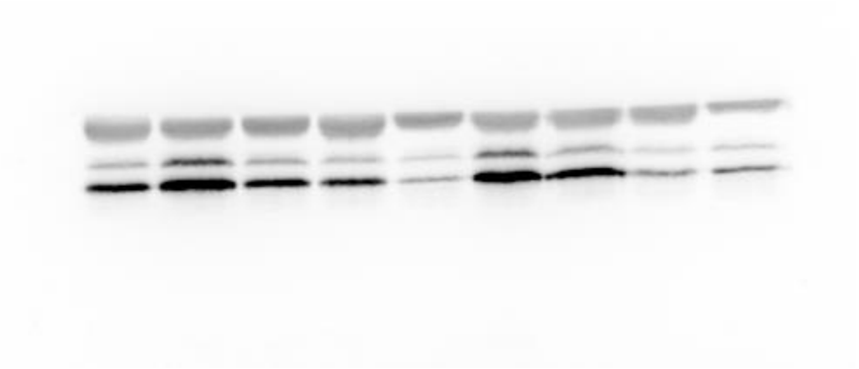

2.DH – total ERK1/2

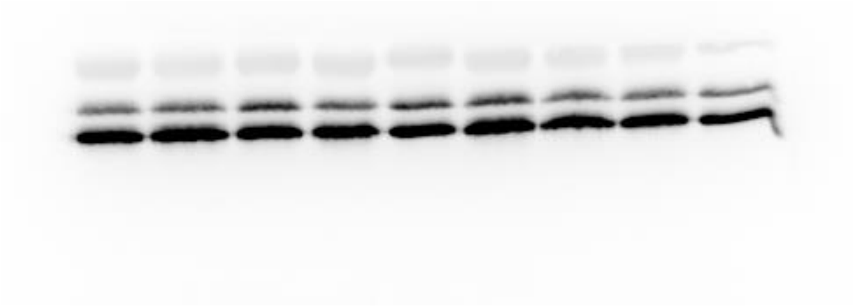

3.DH – Tubulin

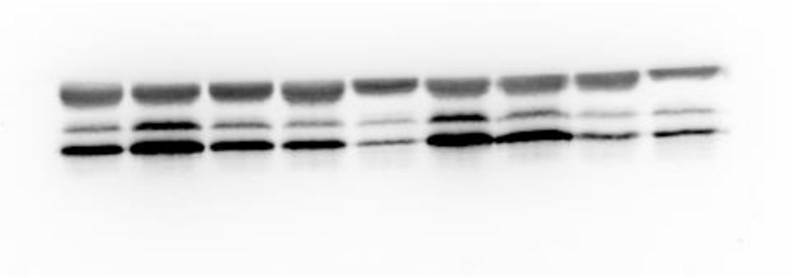

Supplement: Supplementary file 1 [file DataSheet1.PDF]
